# The interkingdom horizontal gene transfer in 44 early diverging fungi boosted their metabolic, adaptive, and immune capabilities

**DOI:** 10.1093/evlett/qrae009

**Published:** 2024-03-05

**Authors:** Michał Aleksander Ciach, Julia Pawłowska, Paweł Górecki, Anna Muszewska

**Affiliations:** Faculty of Mathematics, Informatics and Mechanics, University of Warsaw, Warsaw, Poland; Institute of Biochemistry and Biophysics, Polish Academy of Sciences, Warsaw, Poland; Institute of Evolutionary Biology, Faculty of Biology, Biological and Chemical Research Centre, University of Warsaw, Warsaw, Poland; Faculty of Mathematics, Informatics and Mechanics, University of Warsaw, Warsaw, Poland; Institute of Biochemistry and Biophysics, Polish Academy of Sciences, Warsaw, Poland

**Keywords:** early diverging fungi, horizontal gene transfer, fungal immunity, ecological adaptation, HGT patterns, associated bacteria

## Abstract

Numerous studies have been devoted to individual cases of horizontally acquired genes in fungi. It has been shown that such genes expand the hosts’ metabolic capabilities and contribute to their adaptations as parasites or symbionts. Some studies have provided an extensive characterization of the horizontal gene transfer (HGT) in Dikarya. However, in the early diverging fungi (EDF), a similar characterization is still missing. In order to fill this gap, we have designed a computational pipeline to obtain a statistical sample of reliable HGT events with a low false discovery rate. We have analyzed 44 EDF proteomes and identified 829 xenologs in fungi ranging from *Chytridiomycota* to *Mucoromycota*. We have identified several patterns and statistical properties of EDF HGT. We show that HGT is driven by bursts of gene exchange and duplication, resulting in highly divergent numbers and molecular properties of xenologs between fungal lineages. Ancestrally aquatic fungi are generally more likely to acquire foreign genetic material than terrestrial ones. Endosymbiotic bacteria can be a source of useful xenologs, as exemplified by NOD-like receptors transferred to *Mortierellomycota*. Closely related fungi have similar rates of intronization of xenologs. Posttransfer gene fusions and losses of protein domains are common and may influence the encoded proteins’ functions. We argue that there is no universal approach for HGT identification and inter- and intra-kingdom transfers require tailored identification methods. Our results help to better understand how and to what extent HGT has shaped the metabolic, adaptive, and immune capabilities of fungi.

## Teaser

Horizontal gene transfer is a transmission of genes between unrelated organisms that seems to occur in all living organisms, including fungi. Although several studies focused on the description of the horizontal gene transfer patterns in terrestrial fungi, similar characterization is still missing for early diverging fungi. Through the development of computational pipeline enabling to obtain a statistical sample of reliable horizontal gene transfer events minimizing false positive hits, we demonstrate that it is driven by bursts of gene exchange and duplication, resulting in highly divergent numbers and molecular properties of xenologs between different fungal lineages. These detected gene transfers seem to expand the metabolic, adaptive, and immune capabilities of early diverging fungi.

## Introduction

Horizontal gene transfer (HGT) is a process of exchange of genetic material between organisms. It has been extensively studied in prokaryotes and proved to be an important mechanism of adaptation (Coyte et al., 2022). In eukaryotes, the significance and frequency of HGT remain mostly unclear, and the mechanisms of gene transfer (other than *Agrobacterium*-mediated gene transformation) are still a matter of debate (Naranjo-Ortiz & Gabaldón, 2020). It has been shown, however, that HGT does occur both within eukaryotes (including animals) and between eukaryotes and prokaryotes (Gabaldón, 2020; Keeling & Palmer, 2008).

Existing studies of fungal HGT have mostly focused on the Dikarya fungi (Marcet-Houben & Gabaldón, 2010; Richards et al., 2011), while HGT in the early diverging fungi (EDF, non-Dikarya fungi) is relatively understudied. Recent reports show that EDF experienced several documented waves of transfers, like the acquisition of secondary metabolite clusters in *Basidiobolus* (Tabima et al., 2020), *Mortierella* (Wurlitzer et al., 2021), and *Rhizophagus* (Venice, Desirò, et al., 2020), or enzymes transferred to *Neocallimastigomycota* involved in the metabolism of nucleotides (Alexander et al., 2016) and carbohydrates (Murphy et al., 2019). A more complex scenario was reported for *Rhizophagus irregularis*, which acquired genes both from the host plant and its endosymbiotic bacterium (Li et al., 2018). Catalase genes of bacterial origin, which presumably increase ROS resistance, were found in the amphibian pathogen, *Batrachochytrium dendrobatidis* (Hansberg et al., 2012). However, these results were obtained with different methodologies, and each of them was focused on a selected fungal lineage. There is currently a lack of a comprehensive comparative analysis of HGT between different EDF lineages using a consistent methodology.

In this work, we study the patterns of gene exchange between fungi and nonfungal organisms on a representative set of 44 EDF proteomes ranging from *Chytridiomycota* to *Mucoromycota*. We have designed a custom phylogenetic-based pipeline to obtain a reliable sample of xenologs, focusing on limiting the number of false positive results that distort the observed signal (Irwin et al., 2022), while retaining a sufficient sample size to infer statistical trends. We then analyze the resulting data set of HGTs from a taxonomic, ecological, and molecular perspective. Our results provide a comprehensive characterization of the patterns of HGT across the EDF tree of life.

## Methods

### Dataset

Proteomes of 44 EDF (Supplementary Table S1, sheet assemblies), referred to as the *target proteomes*, were downloaded from NCBI in April 2018 (NCBI Resource Coordinators, 2017). Taxonomical annotations were obtained from the NCBI taxonomy database, with corrections according to (Gabaldón, 2020; Keeling & Palmer, 2008). EDF taxonomy in this manuscript follows Voigt et al. (2021). Genomic features, including intron count, were obtained from the GFF files of the genome assemblies. Assembly completeness was assessed with BUSCO v5 (Manni et al., 2021).

### HGT identification

**Figure 1.**
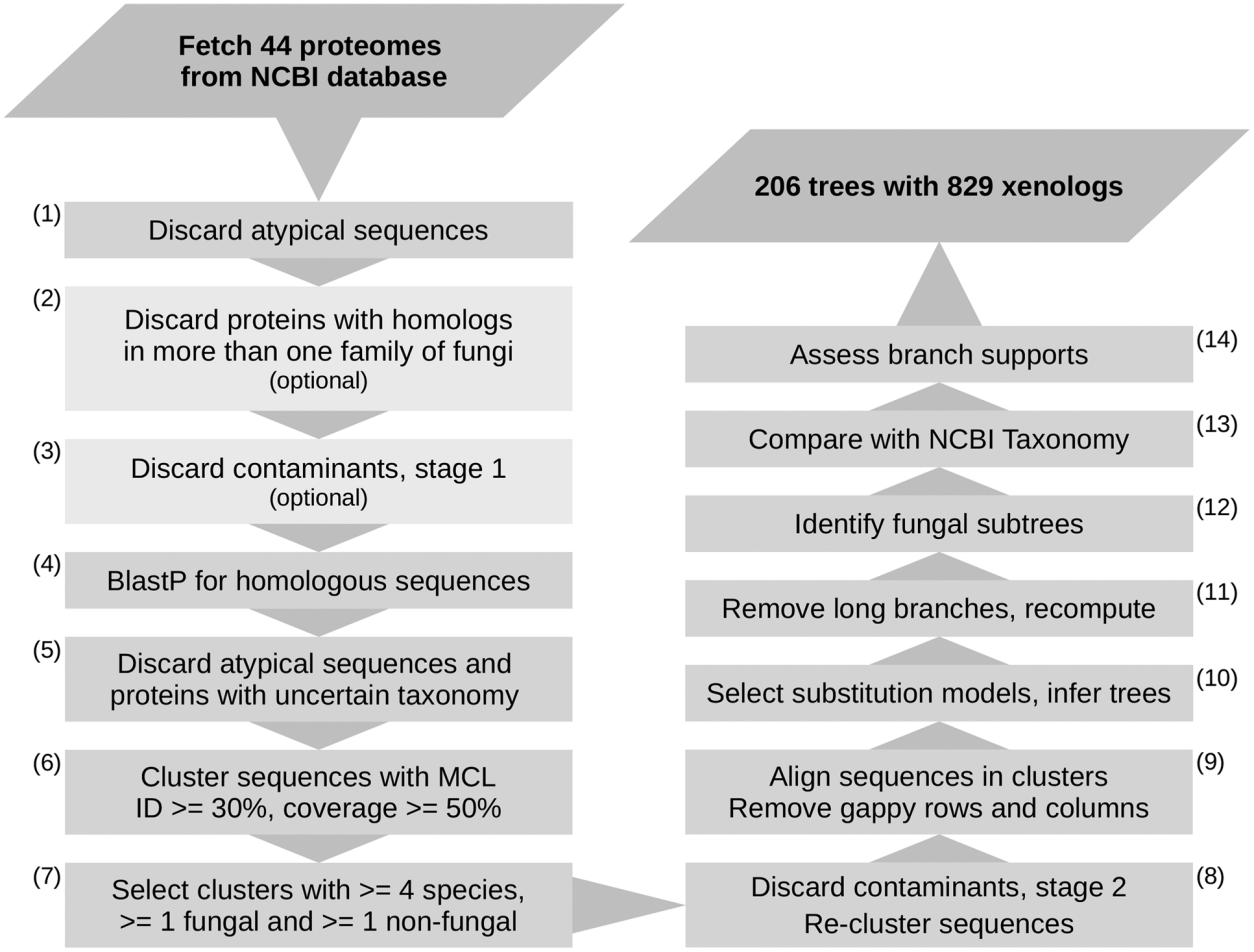
A flow chart of the HGT identification pipeline.

We take a phylogenetic approach where we first detect clusters of protein sequences with a predominantly nonfungal taxonomy (Figure 1, rationale and algorithms Supplementary Methods A–D, commands and scripts; https://github.com/mciach/HGTin44EDF). Next, we calculate gene trees and identify fungal subtrees. We select the subtrees indicative of HGT by comparing the topology of the gene tree against the corresponding species tree from the NCBI Taxonomy database using a custom-made algorithm (Supplementary Methods D). Since the NCBI Taxonomy tree contains many polytomies, we take a conservative approach and report HGTs only if they are supported by all possible binarizations. In our pipeline, we pay a particular attention to separating HGTs from genome contaminations and use a two-step contaminant filtering strategy where we retain only those fungal proteins that are (a) located on a contig with at least one protein-coding gene with a homolog in another fungus and (b) located on a contig with at least one protein-coding gene with a fungal top blast hit. The rationale behind this strategy is discussed in detail in Supplementary Methods A.

### Analysis of xenolog features

Xenolog protein products were mapped against PFAM (El-Gebali et al., 2019) using pfamscan.pl with default parameters (only hits with significance = 1 taken), mapped against KEGG using kofamscan (Aramaki et al., 2020) and scanned for signal peptides with TargetP (Armenteros et al., 2019) and cellular localization with WolfPsort II (Horton et al., 2007). Low-complexity regions were predicted with seg (Wootton & Federhen, 1996) and intrinsically disordered regions with IUPred3 (Erdős et al., 2021). A species tree of the 44 target fungi was inferred from whole proteomes with OMA2 (Altenhoff et al., 2019). To assess the numbers of duplication events after transfer, we have reconciled the fungal subtrees (rooted at the transfer acceptor node) with the OMA2 tree in the duplication-loss model using the ete3 Python package (Page & Charleston, 1997). Statistical and phylogenetic analyses were performed with the R statistical package (https://www.R-project.org/) and the Python 3 programing language using the Jupyter Notebook (Beg et al., 2021) and numpy (Harris et al., 2020), scipy (Virtanen et al., 2020), and ete3 (Huerta-Cepas et al., 2016) libraries. Plots were created using the matplotlib (Bisong, 2019) library. Statistical tests of correlations were performed using a permutation test implemented in the scipy package.

## Results

We have detected 829 xenologs in the 44 target fungi with well-supported phylogenetic evidence for a HGT. The xenologs were nested within 226 subtrees in 206 phylogenetic trees. Including other fungal species, the 226 subtrees contained 1,208 fungal xenologs. For over 50% of trees, iqTree selected the LG model as the best-fitting one, followed by WAG, VT, and JTT models, which jointly accounted for 90% of clusters (Supplementary Figure S1). Nuclear models accounted for 95% of clusters, followed by chloroplast (3%), mitochondrial (1.3%), and viral (0.4%).

Additional 283 trees with 326 fungal subtrees containing 254 target xenologs and 1,857 xenologs from other fungal species had weakly supported or incomplete phylogenetic evidence for HGT and were not used for the statistical analysis of patterns of HGT. The causes for the lack of support are summarized per subtree in Supplementary Figure S1. Note that a single tree may contain multiple fungal subtrees with strong and weak supports for HGT.

Finally, 109 trees contained 123 fungal subtrees with 282 target proteins, which had a congruent location on the gene tree, indicative of a vertical inheritance. Since it is generally considered that topological incongruence between the gene and the species tree is the strongest type of evidence for HGT, we have discarded them from our statistical analyses. However, we have observed a high correlation between the numbers of congruent and incongruent trees per fungal taxon (*ρ* = 0.80, *p* < 1e-12; Supplementary Figure S2).

Supplementary Table S1 stores all identified protein clusters containing fungal xenologs, including unsupported ones (sheet Cluster summary) and a list of well-supported xenologs (sheet Protein summary). In the remaining part of this work, unless explicitly stated otherwise, by fungal subtrees, we understand gene tree subtrees with well-supported phylogenetic evidence for HGT, and by xenologs, we understand proteins from target fungi in those subtrees.

### Genomic results

#### Genome contamination is an order of magnitude more common than HGT in the analyzed proteomes

In this work, we have adopted a two-stage blastp-based contamination filtering. Namely, we discarded contigs without any protein-coding gene with a protein product homologous to any of the other 44 target fungal proteomes and then discarded contigs without a fungal top blast hit.

The first step removed 19,578 proteins (Supplementary Figure S3). This constitutes 3% of the 44 proteomes and 2% of each proteome on average. The number of removed proteins varied highly between proteomes, from 1 in *Absidia repens* NRRL 1336 up to 5,426 in *Rhizophagus irregularis* A1.

The second refined step removed an additional 292 proteins. In most fungi, it removed less than 10 proteins, suggesting that the initial coarse filter was mostly sufficient. The numbers of sequences discarded in both stages were correlated across the 44 proteomes (*ρ* = 0.54, *p* = 0.048). Nevertheless, there was no statistically significant correlation between the numbers of contaminants detected in either stage with BUSCO completeness (*p* = 0.15 for stage 1, *p* = 0.82 for stage 2) or with N50 value (*p* = 0.57 for stage 1, *p* = 0.28 for stage 2).

#### The number of duplications after integration into the host’s genome can be described with a simple mathematical law, which is characterized by infrequent but massive bursts of duplications

**Figure 2.**
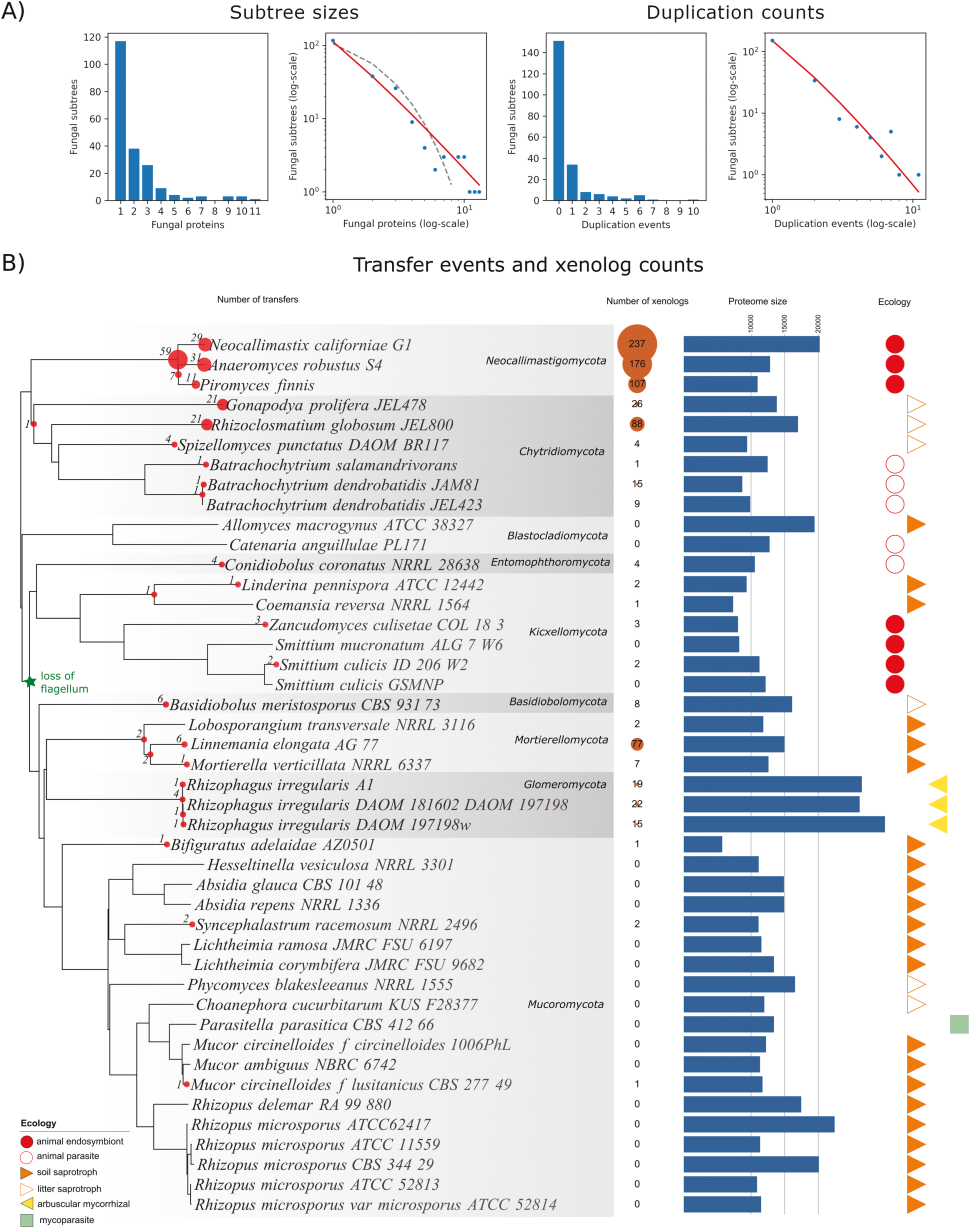
The rates of HGT and posttransfer duplications among the 44 EDF. (A) The fungal subtree sizes (i.e., the numbers of fungal proteins in subtrees originating from HGTs) and the numbers of posttransfer duplications among the target fungi. The red solid lines on the log–log plots correspond to the Yule–Simons distributions (parameter *α* = 1 for subtree sizes, *α* = 2 for duplications); the dashed gray line corresponds to a geometric distribution with parameter 0.47. (B) The OMA2 phylogenetic tree of fungi annotated with the numbers of subtrees of xenologs (brown bubble) and of transfers (red dots, size proportional to the number of events). The loss of flagellum (green star) delineates the ancestrally aquatic fungi from the ancestrally terrestrial ones. Proteome size and ecology of the fungi are given on the right. Taxonomic names based on NCBI Taxonomy with manual fine-tuning according to Voigt et al. (2021). For the position of the outgroup and Dikarya, see Voigt et al. (2021).

**Figure 3.**
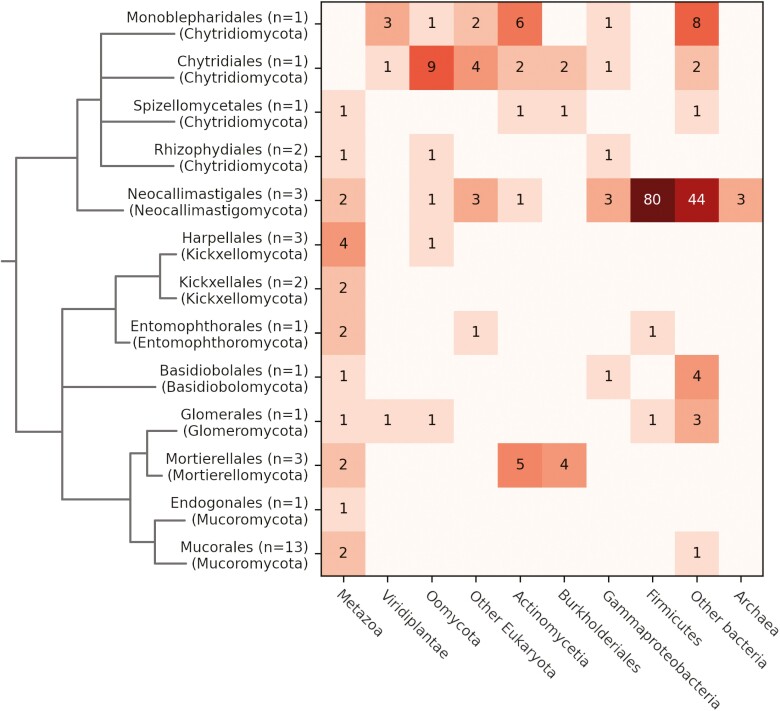
Numbers of fungal subtrees originating from HGTs per acceptor–donor pair. The number of subtrees corresponds to the number of proteins originally transferred to the ancestors of present-day fungi that participated in the exchange of genetic material, i.e., it is the number of xenologs after controlling for paralogy and orthology occurring after transfer. Note that the number of biological events of exchange of genetic material may be lower, as a single exchange can result in transferring multiple proteins.

Over half of the fungal subtrees had only a single leaf, i.e., contained a single fungal protein (Figure 3A). If the numbers of xenologs evolved under a neutral birth-and-death model of gene duplications and losses with similar rates of those events across fungi, the distribution of subtree size would follow a geometric distribution (Nee et al., 1994). However, the chi-square test of goodness of fit performed on subtrees with 1–12 proteins rejects this hypothesis (*p* < 1e-06; ML estimated geometric distribution parameter 0.47). Instead, the distribution of subtree sizes appears to closely follow the Yule–Simon distribution with parameter *α* = 1 (chi-square test *p* = 0.40; Figure 2A), (Udny Yule, 1925; Steel & McKenzie, 2001). This distribution differs qualitatively from the geometric one by having a heavy tail, i.e., noticeable occurrences of large values. This gives a theoretical prediction of massive posttransfer duplications (notably, independent of transposon bursts), and hints at a considerable variance in the posttransfer duplication rates between HGT events. The number of posttransfer duplications among the target fungi also seems to follow a Yule-Simons distribution (chi-square *p* = 0.33 for the distribution parameter *α* = 2; Figure 2A), and so does the number of gene losses.

Characteristically, for heavy-tailed distributions, while 80% of fungal subtrees in our data set contained up to 3 proteins, 1.3% contained more than 50, with the largest one containing 219 xenologs from various *Mucoromycota* species. Since the 219 homologs are contained within a single subtree, they seem to be an example of a massive duplication of foreign genetic material during its vertical evolution after incorporation in the host genome. The target proteins from this subtree contain only an uncharacterized domain (DUF4379); other proteins are involved in DNA repair and recombination (K03746).

#### The number of xenologs is highly divergent across fungi

The number of xenologs in different proteomes was also heavily tailed, with 5 fungal species accounting for over 80% of all detected xenologs (Supplementary Figure S4 and Supplementary Table S1, sheet Organism summary). These included the gut-dwelling *Neocallimastigomycota* (with xenologs constituting more than 1% of their proteomes), the chytrid *Rhizoclosmatium globosum* (0.5%), and the soil saprotrophic *Linnemania elongata* (0.5%). These groups were followed by the plant-symbiotic *Glomeromycota* and parasitic *Chytridiomycota* (with xenologs forming less than 0.2% of their proteomes). We did not observe any statistically significant correlation between the proteome size and the number of xenologs (*ρ* = 0.19, *p* = 0.21). There was no statistically significant correlation of the numbers of detected xenologs with BUSCO completeness (*p* = 0.40), N50 value (*p* = 0.994), nor with the number of contaminating sequences removed in stages 1 (*p* = 0.70) and 2 (*p* = 0.49).

The average proportion of xenologs in proteomes was 0.12%. This figure is within the range reported previously for Dikarya (Marcet-Houben & Gabaldón, 2010).

### Taxonomic results

#### Transfer rates are highly divergent across fungi and do not always reflect the numbers of xenologs in the proteome

We labeled nodes on the phylogenomic tree of the 44 EDFs with the numbers of the corresponding fungal subtrees in our HGT data set (Figure 2B). Note that this procedure only approximates the depth of the transfer in the tree due to possible posttransfer gene losses, incomplete taxon sampling (making the transfer appear more recent), or interfungal transfers after gene acquisition (making the transfer appear more ancient).

There was a clear pattern dividing ancestrally aquatic taxa, with higher HGT rates compared to typical terrestrial fungi. Specifically, the ancestrally aquatic taxa had 80% of xenologs and 90% of xenolog-containing gene trees in our data set, with *Chytridiomycota* and *Neocallimastigomycota* standing out in particular (Figure 2B). The mainly saprotrophic *Mucoromycota* shows the lowest number of HGT events per genome, just as the animal-related *Kickxellomycota* fungi. *Mortierellomycota* and *Glomeromycota* have rather low transfer rates, with posttransfer duplications as the major force driving the number of xenologs in these fungi.

#### Transfer rates reflect physical proximity

Diverse bacteria were the most common donors of xenologs in our data set, including Firmicutes (most prominently Clostridiaceae, Lachnospiraceae, Eubacteriaceae, Oscillospiraceae, and Bacilli), Proteobacteria (γ-proteobacteria, Burkholderiales), and Actinobacteria. These were followed by eukaryotic Oomycota, Metazoa, and Viridiplantae, and only a few transfers from Archaea (Figure 3). In agreement with previous results (Alexandre et al., 2021), in the gut-dwelling *Neocallimastigomycota*, we have detected genes from a myriad of diverse bacteria, including Bacteroidetes, Bacilli (Firmicutes), Slackia (Actinomycetota), and *Aeromonas* (Proteobacteria). Genes from anaerobic Clostridia are said to enable them to adapt to the gut environment (Murphy et al., 2019).

For the soil-inhabiting *Mortierellomycota*, where *Burkholderia*-related endobacteria are common, Burkholderiales stand out as one of the main sources of xenologs. Putative gene donors include *Mycoavidus cysteinexigens* (OAQ22128.1), an endohyphal symbiont of *Linnemannia elongata* (Uehling et al., 2017), and *Mycetohabitans rhizoxinica* (OAQ29846.1), symbionts of *Rhizopus microsporus*. The second main source of transfers to *Mortierellomycota* was the soil-inhabiting Actinomycetia, often associated with fungal isolates (Robinson et al., 2021).

In the water-living *Chytridiomycota*, most xenologs come from loosely associated bacteria such as Alphaproteobacteria, Gammaproteobacteria, and Actinomycetia. Additionally, we have identified several transfers from Oomycota, including a tRNA (N6-threonylcarbamoyladenosine(37)-N6)-methyltransferase TrmO (ORY41869.1) and tandem tetratricopeptide repeat (TPR) proteins (e.g., ORY27572.1). TrmOs are widely present in most domains of life but were apparently lost in fungi. The transfer of the Oomycota gene seems to have compensated for this loss. The TPR proteins are predicted to form a protein-binding surface, but their function remains unknown.

#### *Neocallimastigomycota* received multiple independent transfers of homologous proteins

Out of 206 trees in our data set, 187 contained a single fungal subtree, 18 contained 2, and 1 contained 3. We report two independent transfers of trypsins into *Conidiobolus coronatus* and two transfers of TPR genes to *Rhizoclosmatium globosum* from *Saprolegniaceae*. All the remaining trees with two or three fungal subtrees contained only *Neocallimastigomycota* species. Eight of them corresponded to repeated independent transfers of homologous genes to the ancestral lineage of *Neocallimastigomycota*, e.g., a transfer of a bacterial cellulase once from Bacilli (e.g., *Neocallimastix californiae* G1 ORY56492.1) and once from Clostridia (e.g., *Neocallimastix californiae* G1 ORY21818.1). The remaining trees corresponded to independent transfers directly to different species, e.g., genes with a solute binding domain *SBP_bac_3* transferred to *Neocallimastix californiae* G1 from Oscillospiraceae (ORY20588.1) and to *Anaeromyces robustus* from Methanobacteriaceae (ORX65467.1). The fact that these fungi occupy a similar ecological niche could predispose them to recurrent HGT, resulting in a convergent evolution. This result stands in agreement with a recently reported major HGT event in the ancestor of *Neocallimastigomycota* that might have driven its evolution towards a lifestyle as gut-dwelling fungus of herbivores (Murphy et al., 2019). This result points to a unique feature of *Neocallimastigomycota* genomes in which metabolic redundancy is not only generated through gene duplications, but also through HGT. The fact that repeated transfers were nearly absent in other fungi suggests that the ones detected for *C. coronatus* and *R. globosum* may be artifacts caused by tree inference errors.

### Molecular results

#### Low-complexity sequences are rarely transferred

**Figure 4.**
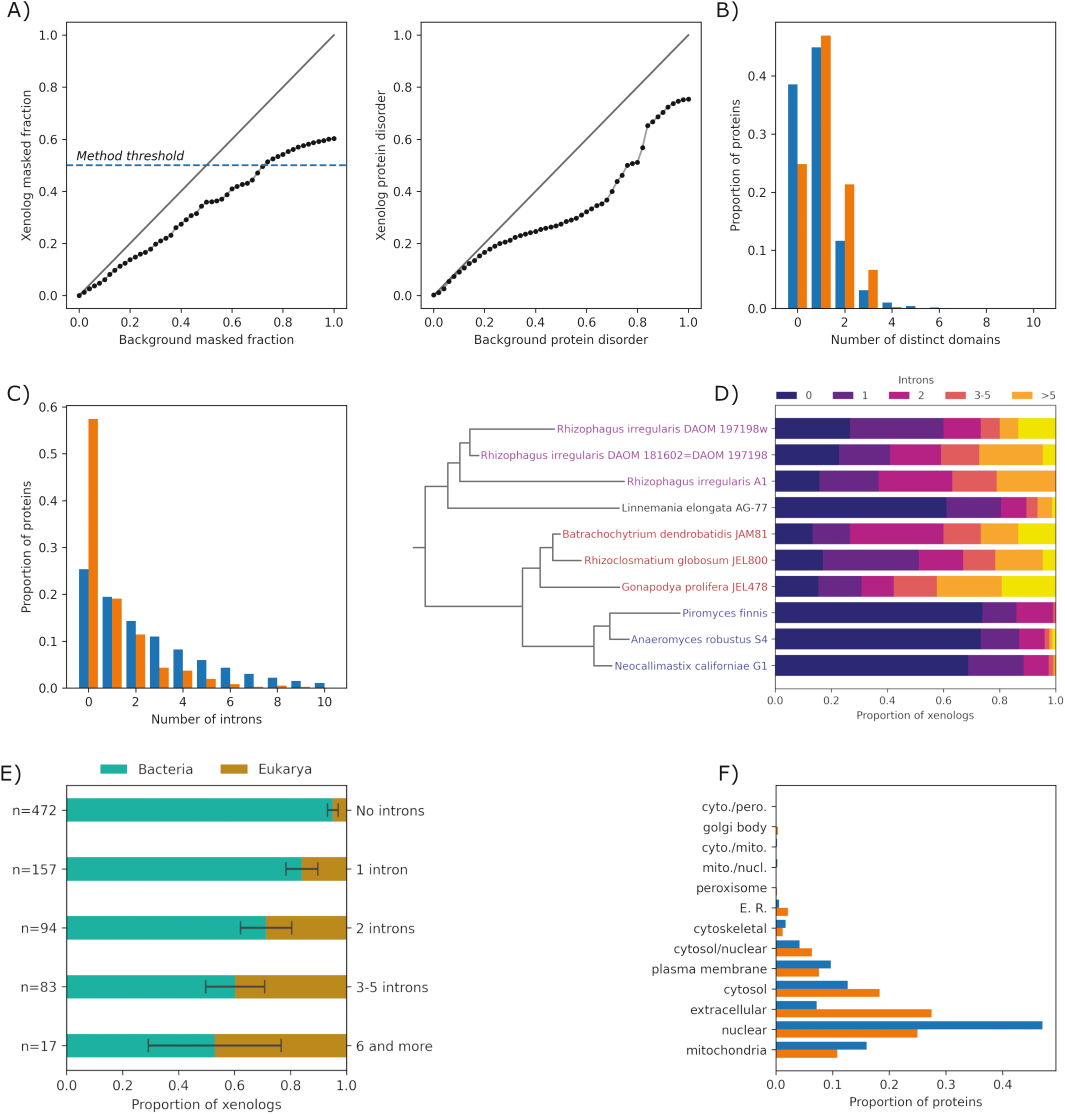
Molecular characteristics of xenolog protein products (orange, right-hand side bars in B, C, bottom bars in F) and background proteins (blue, left-hand side bars in B, C, top bars in F). (A) Quantile plots comparing the xenolog and background sequence low-complexity fraction and intrinsically unstructured fraction. (B) The numbers of identified PFAM domains. (C) The number of introns. (D) A detailed distribution of intron counts in xenologs of selected fungi, showing taxonomy-correlated preferences for closely related fungi. *Glomeromycota* species are highlighted in magenta, Chytrid species in red, *Neocallimastigomycota* species in blue. (E) The number of introns in xenologs and their dependence on the kingdom of the putative donor. (F) The subcellular localizations of xenologs and the background proteins.

The distributions of the proportions of protein sequences covered by low-complexity regions, as well as intrinsically unstructured regions, have lighter tails for xenologs than for the proteome background, visible well below the detection threshold of our method and therefore unlikely to be artifacts of our methodology (Figure 4A). A comparison of the average low-complexity proportions of fungal xenologs to the putative donor group showed no visible trend (Supplementary Figure S5). This suggests that genes encoding proteins with extensive low-complexity or unstructured regions are less likely to be transferred or retained. Note, however, that it may be an artifact caused by impaired homology detection between low-complexity regions (Jarnot et al., 2022).

The distribution of xenolog sequence length follows the background up to around 1,800 amino acids and drops sharply afterward. This seems to be an artifact of our method, which puts a threshold on xenolog sequence length at 2,000 aa. There are documented HGTs of longer sequences, like metabolic clustersHGT (Tabima et al., 2020; Wisecaver et al., 2014). A comparison of the average sequence length of xenologs to the putative donor group showed no visible trend of shortening or lengthening of sequences posttransfer but revealed differences of up to 400 amino acids in both ways (Supplementary Figure S5).

#### Xenologs are predicted to be secreted to the extracellular space more often than typical fungal proteins, but determining their precise subcellular location may be challenging

TargetP 2.0 detected a signal peptide in 33% of identified xenologs, compared to 7.7% of the proteome background. This stands in agreement with the osmotrophic lifestyle of fungi, their massive secretome, and the accessory function of many xenologs. Consistently with the bacterial origin of many xenologs, only nine xenologs had mitochondrial transfer peptides. We did not observe biologically meaningful differences in the numbers of transmembrane helices predicted by TMHMM, although a chi-square test weakly rejects the hypothesis of identical distributions in xenologs and the background (*p* = 0.03, *ddof* = 0, assuming xenolog observations as observed, background as expected).

The subcellular localization predicted by WolfPsort was consistent (i.e., the same for at least 75% of the target fungal proteins in a given subtree) for 77% of subtrees, compared to 95% for TargetP (Supplementary Figure S6). Compared to the proteome background, xenologs were predicted to localize more often in the extracellular space (28% vs. 7%), the endoplasmic reticulum (2% vs. 1%), and the cytoplasm (18% vs. 13%), and less often in the nucleus (25% vs. 47%) and the mitochondria (11% vs. 16%; Figure 4F). We note, however, that these results may be biased toward mitochondrial and nuclear proteins (Supplementary Results).

#### Xenologs typically contain few introns but can acquire many

Over 50% of xenologs did not contain any intron, compared to 25% of the background (Figure 4C). This result is in line with recommended HGT verification measures (Jaramillo et al., 2015). However, we have also detected 17 xenologs with 6 or more introns (Figure 4E). In particular, the *Rhizophagus irregularis*, *Gonapodya prolifera*, and *Batrachochytrium dendrobatidis* were prone to have intron-rich xenologs from both unicellular eukaryotic and putative prokaryotic donors. The preference for the intron structure of xenologs seemed correlated taxonomically: while the xenologs in *Chytridiomycota* tended to contain multiple introns, the ones in *Neocallimastigomycota* tended to contain few, if any (Figure 4D). However, the correlation breaks rapidly at higher taxonomic levels.

The number of introns closely follows a geometric distribution in the fungal proteome background but not in the set of xenologs. In particular, the distribution in xenologs was zero-inflated, consistent with the bacterial origin of many xenologs. However, many bacteria-derived xenologs contained multiple introns (Figure 4E), suggesting that they acquire introns after the transfer, consistent with previous reports (Lage et al., 2013). This seemed to be the case in an *R*hizoclosmatium* globosum* xenolog with twelve introns (ORY15655.1) derived from a deltaproteobacterium *Labilithrix luteola* magnesium transporter. This protein had three posttransfer paralogs, out of which one contained only one intron, one contained five, and one contained six (respectively ORY29294.1, ORY29292.1, and ORY15656.1), suggesting an ongoing intronization process. A similar scenario seems to have shaped the story of arginine decarboxylase biosynthesis gene, which was transferred from an intronless gene of an anaerobic deltaproteobacterium *Anaeromyxobacter dehalogenans* (locus_tag A2cp1_2068) and acquired nine introns after transfer to *R. globosum* (KXS13513.1). In addition to the intronization of bacteria-derived xenologs, we have also observed an apparent intron gain in an E3 ubiquitin–protein ligase gene with 8 introns and transferred from some early diverged metazoans to *Glomeromycota* (EXX59914.1). The closest metazoan homologs from *Acropora millepora* (LOC114972538) and *Orbicella faveolata* (LOC110047255) have only three or four introns.

To test the hypothesis of intron acquisition in bacteria-derived xenologs, we have compared the number of introns to the branch distance to the root of the corresponding fungal subtree (i.e., the posttransfer number of substitutions per site). A linear model fitted without an intercept estimated 0.6 introns per unit branch length, i.e., per one substitution per site (*p* < 0.001). However, the overall picture is more complicated, as some xenologs quickly acquired multiple introns, while many others had none even after a long time (Supplementary Figure S7).

#### Gene transfer seems to frequently involve acquiring and losing protein domains

Compared to the proteome background, fungal sequences obtained via HGT were more likely to map to one or more distinct PFAM domains (Figure 4B). The average number of domains was 0.85 for the background and 1.11 for the xenologs. However, 100 fungal xenologs had domains that had no clan-level siblings in nonfungal sequences from the same gene tree and were therefore apparently acquired during or after the incorporation of the foreign genetic material in the host genome. Most xenologs acquired a single domain, while 10 acquired two distinct domains. Except for one xenolog in *Linnemania elongata* and five xenologs in chytrids, all the domain acquisitions happened in *Neocallimastigomycota*. As therefore expected, the by far most commonly added domain was the carbohydrate-binding module CBM_10 (PF02013), a part of the dockerin cellulosome typical of anaerobic fungi, acquired in 86 xenologs. Consistent with the function of CBM_10, this domain was fused preferentially to xenologs containing domains associated with carbohydrate metabolism, such as the bacterial glycosyl hydrolase (PF02011) in 31 xenologs. A similar case involved 13 xenologs with a bacterial CotH kinase domain transferred to *Neocallimastigomycota*. These CotH proteins, likewise in bacteria, may play a role in plant cell wall binding (Haitjema et al., 2017). The next most commonly acquired domain was the ricin_B_lectin domain (PF14200) acquired in nine *Neocallimastigomycota* xenologs.

Some domains were seemingly lost during transfer, with 89 xenologs lacking domains which were present in at least 20% of sequences in both neighboring gene tree subtrees. An example was a bacteria-derived cellulase precursor ORX60185.1 in *Piromyces finnis*, which was missing an N-terminal fragment of its bacterial homologs with a cellulose binding domain CBM_2 and a Bacterial Ig domain Big_7. On the other hand, this protein contained a bacterial glycoside hydrolase domain Glyco_hydro_48 and was posttransfer fused with a cellulose binding domain CBM_10. Effectively, during or after the incorporation of a foreign genetic material, a bacterial carbohydrate-binding domain was replaced with a fungal one.

Overall, of 829 xenologs detected in our target fungi, 170 had a novel or a lost domain, and 48 had both (such as the aforementioned cellulase precursor in *P. finnis*). This agrees with the differences in protein sequence lengths up to 400 aa between the acceptors and donors discussed above. In some cases, this resulted in conflicting phylogenetic signals. The *R. globosum* ORY15655.1 (one of the aforementioned intron-rich bacteria-derived xenologs) contained a xenologous N-terminal region of 530 aa of bacterial origin with a glycoside hydrolase domain (InterPro *Glycoside_hydrolase_SF*) and a C-terminal region of 180 aa apparently inherited vertically, with a mainly eukaryotic magnesium transporter NIPA domain Mg_trans_NIPA. We note that this apparent gene fusion may have simply been a gene calling error merging two neighboring genes, and further proof is required for confirmation. Regardless of the actual source of the apparently fungal region, the presence of conflicting signals in this protein’s sequence severely impacts the topology and support of the phylogenetic tree (Supplementary Figure S8).

**Figure 5.**
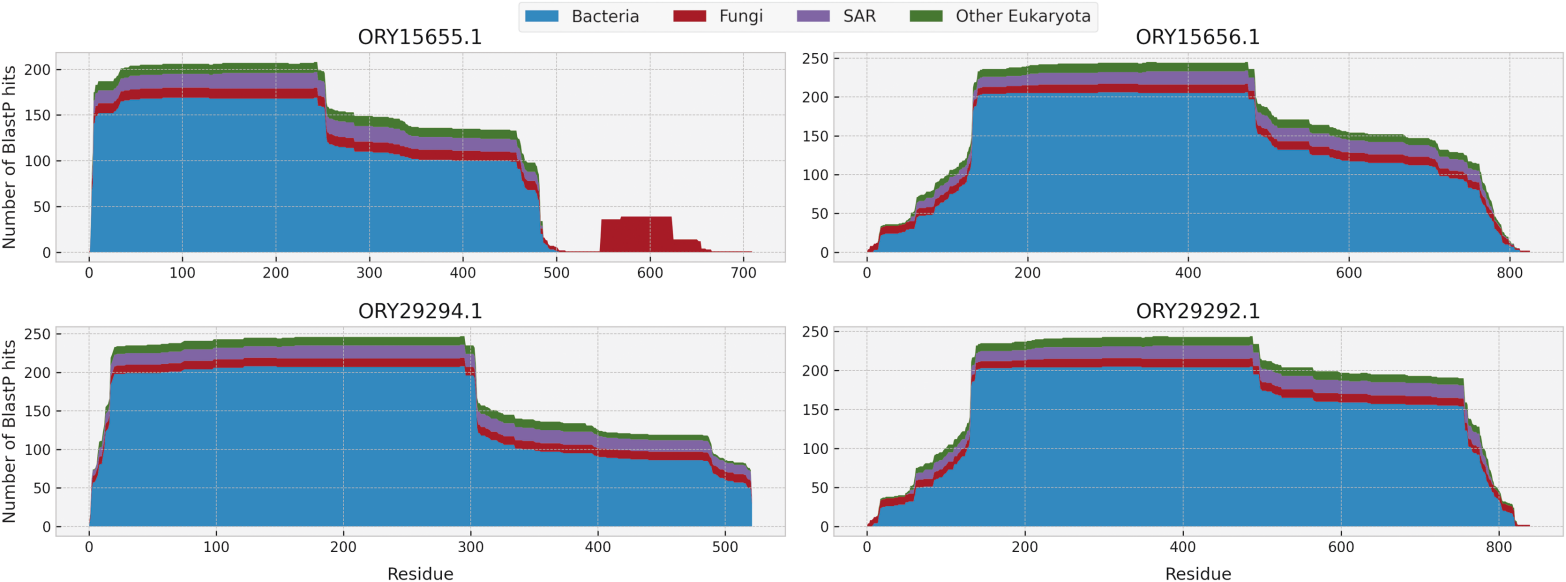
A posttransfer fusion of a fungal domain with a xenolog. Plots visualize the taxonomic composition of the first 250 BlastP hits per amino acid of the query protein. The ORY15655.1 protein contains a distinct C-terminal region, which appears to be inherited vertically and contains a magnesium transporter domain. The posttransfer paralogs of this protein (ORY15656.1, ORY29294.1, ORY29292.1) have a similar taxonomic distribution of their BlastP hits at each amino acid but lack the domain. These paralogs have no PFAM or InterPro domains, suggesting a possible change of function after duplication. See Supplementary Figure S9 for an alignment of the four proteins and the location of the domains in ORY15655.1.

Notably, the 12 introns of the *R. globosum* ORY15655.1 xenolog were located in both the fungal and the bacteria-derived region. The three aforementioned putative posttransfer paralogs of this protein with fewer introns (ORY29294.1, ORY29292.1, and ORY15656.1) lack the apparently fungal C-terminal region (Figure 5). Whether the presence of a fungal genetic material has facilitated the intronization of ORY15655.1 is an open question. Interestingly, the three paralogs also lack any PFAM or InterPro domains, including the bacteria-derived InterPro glycoside hydrolase domain. On the other hand, two paralogs (ORY15656.1 and ORY29292.1) contain a bacterial N-terminal region which appears to have been lost in ORY15655.1 and ORY29294.1 (Supplementary Figure 9). This indicates a complex evolution of the gene after incorporation in the fungal genome, involving duplication of the foreign genetic material, domain gain and loss in the paralogs, and an ongoing intronization.

#### Metabolic domains constitute the majority of xenologs

We have selected domains that were present in at least half of the fungal proteins in a given subtree and in at least 20% of proteins in one of the neighboring subtrees (Supplementary Table S1). Domains of unknown function (DUFs/UPFs) constituted numerous functional classes with 16 diverse Pfam families found in 60 xenologs. Among them are those involved in metabolism, particularly coding alpha/beta hydrolases, acetyltransferases, and esterases. Many carbohydrate-active enzymes (a total of 58 CaZymes, glycohydrolases, and glycosyltransferases), carbohydrate-binding lectins (*n* = 7), and cellulases (*n* = 48) were transferred to *Neocallimastigomycota*, in agreement with (Murphy et al., 2019). Peptidases were underrepresented in the dataset (peptidase xenologs n = 24) compared to the CaZymes and carbohydrate-binding modules (*n* = 63), but were transferred to diverse recipients. They included trypsins transferred from a bacterium *Phytohabitans flavus* to *Spizellomyces punctatus* (KNC97026.1) and from a spider *Araneus ventricosus* to *Conidiobolus coronatus* (KXN68345.1), and a membrane-bound dipeptidase, metalloproteinase M19, transferred from Micromonosporaceae bacteria to *G. prolifera* (KXS08983.1). Trypsin expansions can be linked to enhanced degradation of protein-rich substrates such as insects, while a M19 peptidase is a part of an *Aspergillus* spp. gliotoxin biosynthesis cluster.

The dominance of CaZymes over peptidases can be linked to the high number of transfers to *Neocallimastigomycota*. These fungi also acquired genes with a myriad of specificities connected to secondary metabolism and transport, extending their nutritional capabilities. Condensation domain (PF00668) containing candidate NRPS secondary metabolite clusters were transferred from Eubacteriales to several *Neocallimastigomycota* (e.g., *Anaeromyces robustus* ORX81431.1). General transporters from MFS_1 and ABC families were also acquired by these fungi (e.g., ORY49205.1).

HGT has likely contributed to both fungal immunity and pathogenicity. A bacterial putative betalactamase resistance gene was transferred to *G. prolifera* JEL478 (KXS12969.1). *Mortierellomycota* acquired NOD-like receptors with NACHT-PPR-WD40 architecture from *Mycoavidus cysteinexigens* endosymbionts. NOD-like receptors are suspected to play a role in Dikarya immunity (Dyrka et al., 2014, Uehling et al., 2017). Interestingly, all of the *Mortierellomycota* NOD-like receptors in our dataset seem to be of a bacterial origin or at least shared with the endosymbiont. *Mycoavidus* homologs have ~50%–45% of sequence identity and are the best reciprocal blast hits. It is an open question whether these proteins have a role in the symbiotic interaction.

On the other side, we have detected transfers of several putative toxins, such as bacterial ricin-type sugar binding modules (*Neocallimastigomycota*, 16 xenologs in a single subtree; e.g., ORX83988.1, ORY77981.1, and ORX84139.1, ORY73345.1 in another tree), associated with bacterial glycoside hydrolase domains and nearly all posttransfer fused with CBM_10 domain. Similar proteins were recently documented in *Mucor* acting as toxins (mucoricin) (Soliman et al., 2021). Among other examples, a sea anemone pore-forming toxin (PF06369) was transferred from a lycophyte plant *Selaginella moellendorffii* to *R. irregularis* and was present in all three strains analyzed in this study (e.g., PKC66727.1).

## Discussion

Our aim was to provide a statistical characterization of the patterns of interkingdom HGT across diverse EDF. In this kind of high-throughput study, there are multiple factors that can inflate the false positive rate of HGT detection (see Supplementary Methods B for a detailed discussion), including, but not limited to:

- Relying on sequence similarity instead of phylogenetic trees (Koski & Brian Golding, 2001),
- Excessively long, multidomain proteins, sometimes originating from ORF prediction errors (Poptsova & Gogarten, 2010),
- Multiple conflicting phylogenetic signals in a single protein caused by gene fusion or gene calling error (as shown in this work),
- Sequences without a well-defined location in the species tree (e.g., viral or environmental),
- Low-quality alignments and a wrong choice of the trimming software (Tan et al., 2015),
- Automatic rooting of gene trees (Lamarca et al., 2022; Wade et al., 2020),
- Low-quality gene trees with low support values and long branches (Rodríguez-Ezpeleta et al., 2007),
- Using tree reconciliation algorithms with improper parameters (Libeskind-Hadas et al., 2014).

Our results suggest that the false positive risk factors listed above have a greatly varying impact on the quality of the results. Moreover, while there has been much research devoted to some, others seem to be underappreciated in the literature. Phylogenetic tree incongruence is widely considered the gold standard of HGT detection and has been the basis of our approach. However, when considering phylogenetic trees of homologs of fungal proteins with an atypical taxonomic distribution, the high correlation between the numbers of congruent and incongruent trees per fungal taxon suggests that taxonomy-based methods such as the Alien Index (Gladyshev et al., 2008; Rancurel et al., 2017) may be equally reliable. The added benefit of phylogeny-based methods is a more precise determination of the putative gene donors, but it is important to note that this precision is always limited by the completeness of the available databases, and the identified putative donors are at best, only the closest sequenced relatives of the actual donors.

While tree-based methods seem to offer only a marginal improvement over taxonomy-based heuristics, the filtering of contaminant sequences is of tremendous importance. In EDF, genome contamination may occur due to sample contamination or the presence of endohyphal bacteria. We have detected nearly 20,000 likely contaminating sequences, 20 times more than the number of detected xenologs. As a consequence, a randomly selected gene with an atypical taxonomy of homologs or an incongruent phylogeny is much more likely to be a contaminant than a xenolog. The ultimate determination of whether a gene is incorporated into the genome and expressed requires experimental evidence; in bioinformatic analysis, the minimum requirement should be the presence of genes with evidence of vertical inheritance in the same contig, especially since approaches based on sequence identity and GC content may be insufficient to separate xenologs from contaminants. The picture is further complicated by the fact that fungal endosymbionts are still understudied, and most likely, there still are many unknown endohyphal organisms (Robinson et al., 2021).

We have shown that one of the most predictable features of HGT in EDF is its unpredictability. The number of xenologs in a given proteome is driven by at least two burst-like events: bursts of gene exchange like in *Neocallimastigomycota* and bursts of gene duplications like in *Linnemania* sp. This agrees with unexpectedly high numbers of xenologs in some metazoan proteomes, like *Rotifera* sp. (Gladyshev et al., 2008). Consequently, there seem to be multiple different mechanisms in which a fungal genome can become enriched in xenologs, and each of those mechanisms occurs to a different extent in different fungi. *Linnemannia elongata* rarely participated in gene exchange but multiplied some of the received genetic material extensively, resulting in 77 xenologs in 10 gene trees; *Gonapodya prolifera* JEL478 participated in multiple gene exchanges, but the received material was rarely duplicated, resulting in 26 xenologs in 22 gene trees; *Rhizoclosmatium globosum* JEL800 both readily participated and multiplied some of the received genes, resulting in 88 xenologs in 22 gene trees; and the ancestor of *Neocallimastigomycota* participated in genetic exchange to the extent that it received multiple copies of related proteins from different, unrelated donors. The complexity increases as a single genetic exchange event can result in transferring multiple genes that encode proteins from various families. Nevertheless, despite the different lineage-specific ways of enriching a genome in foreign genetic material, we have also identified some general patterns in the properties of protein products of horizontally transferred genes.

In agreement with the current notions, the xenologs in our dataset tend to contain few introns except for chytrids and some *Glomeromycota*. This seems to have resulted from both the acquisition of intron-rich genes and the intronization of xenologs. The distribution of introns seems to be taxonomy-dependent, with certain fungal groups showing a preference for intronless xenologs while others for intron-rich ones. Bacteria-derived xenologs acquire introns over time, with some being particularly enriched in introns, while others being particularly resistant to intronization or not yet ameliorated to the fungal genome.

The receptiveness for foreign genes varies greatly between fungal lineages. In agreement with previous reports, we have found that fungi belonging to ancestrally aquatic groups experienced more HGT events than terrestrial lineages. This can be explained by the easier exchange of genetic material in the aquatic ecosystem, the ubiquity of unicellular aquatic life forms, and the possibility of forming biofilms with bacteria (Abe et al., 2020; Grüll et al., 2018; McDaniel et al., 2010). Terrestrial fungi may have lost some of the HGT predisposing traits or gained defensive mechanisms preventing foreign gene incorporation. However, the relationship with the host and the necessity to adapt to a new environment seem to have a higher influence on the rate of HGT. Our observations suggest that, while symbiosis or parasitism does not always result in gene exchange, it is a predisposing condition.

Some of the early diverging fungal lineages have associated bacteria, either endohyphal or loosely related to their hyphae, some of which are co-evolving with the fungal host and transmitted vertically (Pawlowska et al., 2018). Those bacteria have been previously pointed to as a likely source of xenologs (Naito et al., 2015). This pattern was confirmed in this study, with a notable example of *Podila verticillata* acquiring fatty acid desaturases probably from deltaproteobacteria and NOD-like receptors from endohyphal *Burkholderia*-related endosymbiont *Mycoavidus* sp. The latter suggests a parallel evolution of NOD-like receptors and perhaps a NOD-like receptor-based immunity in Dikarya and *Mortierellomycota*. Moreover, NOD-like receptors may be involved in the host-bacteria interaction. A parallel case of HGT of NOD-like receptors was reported in *Gigasporaceae* and expressed in response to *Gigaspora* endosymbiont CaGg (Venice et al., 2020).

Out of 829 xenologs detected in our target fungi, 100 had apparently posttransfer fused domains. This may point to an unexpectedly high frequency of gene fusions occurring during or after the integration of foreign DNA. Cross-kingdom HGT has been reported previously as an important factor in gene fusion (Yanai et al., 2002). As the foreign genetic material is incorporated into the host’s genome, it can be located within an existing gene. Alternatively, homologous or nonhomologous recombination can occur after the new gene is incorporated, especially if it contains a fragment similar to the host’s genes. While it is unclear which mechanism was responsible for the swapping of the bacterial cellulose binding domain with a fungal one in *Piromyces finnis* ORX60185.1, a replacement of a domain that preserves the protein’s function agrees with the current notions about the evolution of domain architectures.

Note that the 100 fusions were detected only based on identified domains; since many sequences lack identified domains, the full extent of posttransfer gene fusion is unknown. Frequent fusions may significantly complicate the studies of HGT. Recombination of transferred and inherited genetic material may, on one hand, confound the impact of HGT on the host’s ecology and evolution, and on the other hand, the proteins’ phylogenetic signals. While most research to date, including this one, has focused on detecting “whole-protein” xenologs, our results indicate the need to develop domain-level methods of xenology detection. This agrees with the recent ideas that although terms like orthology, paralogy, or xenology are traditionally applied on a level of whole genes or proteins, they should perhaps also be considered on a domain-level basis (Forslund et al., 2018; Linard et al., 2021; Persson et al., 2019; Sjölander et al., 2011). We also note that sequence regions with independent evolutionary histories are another reason why the first blast hit may not be the phylogenetically closest neighbor, as a small but highly conserved region may have a lower *E*-value than a large but less conserved one.

Our methodology has detected a number of xenologs reported in the literature (see Supplementary Methods B). However, due to the stringent filtering criteria required to minimize the number of false detections, it also has certain limitations, and the list of xenologs reported in this work is far from exhaustive. In particular, it does not detect transfers between fungal species and may have a limited sensitivity toward transfers of animal genes, many of which have a fungal homolog. Consequently, while our results confirm the occurrence of cross-kingdom gene transfers between eukaryotes (Richards et al., 2009; Schönknecht et al., 2014; Van Etten & Bhattacharya, 2020), they may underestimate their prevalence. In conclusion, although much research has already been done, a thorough characterization of the rates and patterns of HGT still requires extensive further work in optimizing HGT detection protocols and algorithms.

## Supplementary Material

qrae009_suppl_Supplementary_Tables

qrae009_suppl_Supplementary_Figures

## Data Availability

Phylogenetic trees of supported and unsupported HGT along the HGT detection pipeline are deposited under https://doi.org/10.5281/zenodo.10649525; all accessions and assemblies are listed in Supplementary Table S1.

## References

[CIT0001] Abe, K., Nomura, N., & Suzuki, S. (2020). Biofilms: Hot spots of horizontal gene transfer (HGT) in aquatic environments, with a focus on a new HGT mechanism. FEMS Microbiology Ecology, 96(5), fiaa031. 10.1093/femsec/fiaa031 32109282 PMC7189800

[CIT0002] Alexander, W. G., Wisecaver, J. H., Rokas, A., & Hittinger, C. T. (2016). Horizontally acquired genes in early-diverging pathogenic fungi enable the use of host nucleosides and nucleotides. Proceedings of the National Academy of Sciences of the United States of America, 113(15), 4116–4121. 10.1073/pnas.1517242113 27035945 PMC4839431

[CIT0003] Alexandre, F. S., Della Flora, L. V., Henrique, I. G., da Silva, D. C., Mercedes, A. P., Silva, A. C., de Oliveira, A. S., da Silva, M. P. B., Ronning, B. P. F., Dreher, D. R., Cano, B. G., de Lima Andreata, M. F., Filho, J. B., Santos, E. R., Takisawa, F. H., Alfenas, R. F., Andrade, G., & Cely, M. V. T. (2021). *Arbuscular mycorrhizal* fungi (*Rhizophagus clarus*) and Rhizobacteria (*Bacillus subtilis*) can improve the clonal propagation and development of teak for commercial plantings. Frontiers in Plant Science, 12, 628769. 10.3389/fpls.2021.628769 34276714 PMC8284393

[CIT0004] Altenhoff, A. M., Levy, J., Zarowiecki, M., Tomiczek, B., Warwick Vesztrocy, A., Dalquen, D. A., Müller, S., Telford, M. J., Glover, N. M., Dylus, D., & Dessimoz, C. (2019). OMA standalone: Orthology inference among public and custom genomes and transcriptomes. Genome Research, 29(7), 1152–1163. 10.1101/gr.243212.118 31235654 PMC6633268

[CIT0005] Aramaki, T., Blanc-Mathieu, R., Endo, H., Ohkubo, K., Kanehisa, M., Goto, S., & Ogata, H. (2020). KofamKOALA: KEGG Ortholog assignment based on profile HMM and adaptive score threshold. Bioinformatics, 36(7), 2251–2252. 10.1093/bioinformatics/btz859 31742321 PMC7141845

[CIT0006] Armenteros, J. J. A., Tsirigos, K. D., Sønderby, C. K., Petersen, T. N., Winther, O., Brunak, S., von Heijne, G., & Nielsen, H. (2019). SignalP 5.0 improves signal peptide predictions using deep neural networks. Nature Biotechnology, 37(4), 420–423. 10.1038/s41587-019-0036-z 30778233

[CIT0007] Beg, M., Taka, J., Kluyver, T., Konovalov, A., Ragan-Kelley, M., Thiery, N. M., & Fangohr, H. (2021). Using Jupyter for reproducible scientific workflows. Computing in Science & Engineering, 23(2), 36–46. 10.1109/mcse.2021.3052101

[CIT0008] Bisong, E. (2019). Matplotlib and seaborn. In Building machine learning and deep learning models on google cloud platform. Apress, Berkeley, CA. (pp. 151–165). 10.1007/978-1-4842-4470-8_12

[CIT0009] Coyte, K. Z., Stevenson, C., Knight, C. G., Harrison, E., Hall, J. P. J., & Brockhurst, M. A. (2022). Horizontal gene transfer and ecological interactions jointly control microbiome stability. PLoS Biology, 20(11), e3001847. 10.1371/journal.pbio.3001847 36350849 PMC9678337

[CIT0010] Dyrka, W., Lamacchia, M., Durrens, P., Kobe, B., Daskalov, A., Paoletti, M., Sherman, D. J., & Saupe, S. J. (2014). Diversity and variability of NOD-like receptors in fungi. Genome Biology and Evolution, 6(12), 3137–3158. 10.1093/gbe/evu251 25398782 PMC4986451

[CIT0011] El-Gebali, S., Mistry, J., Bateman, A., Eddy, S. R., Luciani, A., Potter, S. C., Qureshi, M., Richardson, L. J., Salazar, G. A., Smart, A., Sonnhammer, E. L. L., Hirsh, L., Paladin, L., Piovesan, D., Tosatto, S. C. E., & Finn, R. D. (2019). The Pfam protein families database in 2019. Nucleic Acids Research, 47(D1), D427–D432. 10.1093/nar/gky995 30357350 PMC6324024

[CIT0012] Erdős, G., Pajkos, M., & Dosztányi, Z. (2021). IUPred3: Prediction of protein disorder enhanced with unambiguous experimental annotation and visualization of evolutionary conservation. Nucleic Acids Research, 49(W1), W297–W303. 10.1093/nar/gkab408 34048569 PMC8262696

[CIT0013] Forslund, K., Pereira, C., Capella-Gutierrez, S., da Silva, A. S., Altenhoff, A., Huerta-Cepas, J., Muffato, M., Patricio, M., Vandepoele, K., Ebersberger, I., Blake, J., Fernández Breis, J. T., Boeckmann, B., Gabaldón, T., Sonnhammer, E., Dessimoz, C., & Lewis, S.; Quest for Orthologs Consortium. (2018). Gearing up to handle the mosaic nature of life in the quest for orthologs. Bioinformatics, 34(2), 323–329. 10.1093/bioinformatics/btx542 28968857 PMC5860199

[CIT0014] Gabaldón, T. (2020). Patterns and impacts of nonvertical evolution in eukaryotes: A paradigm shift. Annals of the New York Academy of Sciences, 1476(1), 78–92. 32860228 10.1111/nyas.14471 PMC7589212

[CIT0015] Gladyshev, E. A., Meselson, M., & Arkhipova, I. R. (2008). Massive horizontal gene transfer in bdelloid rotifers. Science, 320(5880), 1210–1213. 10.1126/science.1156407 18511688

[CIT0016] Grüll, M. P., Mulligan, M. E., & Lang, A. S. (2018). Small extracellular particles with big potential for horizontal gene transfer: Membrane vesicles and gene transfer agents. FEMS Microbiology Letters, 365(19), fny192. 10.1093/femsle/fny192 30085064

[CIT0017] Haitjema, C. H., Gilmore, S. P., Henske, J. K., Solomon, K. V., de Groot, R., Kuo, A., Mondo, S. J., Salamov, A. A., LaButti, K., Zhao, Z., Chiniquy, J., Barry, K., Brewer, H. M., Purvine, S. O., Wright, A. T., Hainaut, M., Boxma, B., van Alen, T., Hackstein, J. H. P., … O'Malley, M. A. (2017). A parts list for fungal cellulosomes revealed by comparative genomics. Nature Microbiology, 2, 17087. 10.1038/nmicrobiol.2017.87 28555641

[CIT0018] Hansberg, W., Salas-Lizana, R., & Domínguez, L. (2012). Fungal catalases: Function, phylogenetic origin and structure. Archives of Biochemistry and Biophysics, 525(2), 170–180. 10.1016/j.abb.2012.05.014 22698962

[CIT0019] Harris, C. R., Millman, K. J., van der Walt, S. J., Gommers, R., Virtanen, P., Cournapeau, D., Wieser, E., Taylor, J., Berg, S., Smith, N. J., Kern, R., Picus, M., Hoyer, S., van Kerkwijk, M. H., Brett, M., Haldane, A., Del Río, J. F., Wiebe, M., Peterson, P., … Oliphant, T. E. (2020). Array programming with NumPy. Nature, 585(7825), 357–362. 10.1038/s41586-020-2649-2 32939066 PMC7759461

[CIT0020] Horton, P., Park, K. -J., Obayashi, T., Fujita, N., Harada, H., Adams-Collier, C. J., & Nakai, K. (2007). WoLF PSORT: Protein localization predictor. Nucleic Acids Research, 35(Web Server issue), W585–W587. 10.1093/nar/gkm259 17517783 PMC1933216

[CIT0021] Huerta-Cepas, J., Serra, F., & Bork, P. (2016). ETE 3: Reconstruction, analysis, and visualization of phylogenomic data. Molecular Biology and Evolution, 33(6), 1635–1638. 10.1093/molbev/msw046 26921390 PMC4868116

[CIT0022] Irwin, N. A. T., Pittis, A. A., Richards, T. A., & Keeling, P. J. (2022). Systematic evaluation of horizontal gene transfer between eukaryotes and viruses. Nature Microbiology, 7(2), 327–336. 10.1038/s41564-021-01026-3 34972821

[CIT0023] Jaramillo, V. D. A., Sukno, S. A., & Thon, M. R. (2015). Identification of horizontally transferred genes in the genus *Colletotrichum* reveals a steady tempo of bacterial to fungal gene transfer. BMC Genomics, 16(1), 2. 25555398 10.1186/1471-2164-16-2 PMC4320630

[CIT0024] Jarnot, P., Ziemska-Legiecka, J., Grynberg, M., & Gruca, A. (2022). Insights from analyses of low complexity regions with canonical methods for protein sequence comparison. Briefings in Bioinformatics, 23(5), bbac299. 10.1093/bib/bbac299 35914952 PMC9487646

[CIT0025] Keeling, P. J., & Palmer, J. D. (2008). Horizontal gene transfer in eukaryotic evolution. Nature Reviews Genetics, 9(8), 605–618. 10.1038/nrg2386 18591983

[CIT0026] Koski, L. B., & Brian Golding, G. (2001). The closest BLAST hit is often not the nearest neighbor. Journal of Molecular Evolution, 52(6), 540–542. 10.1007/s002390010184 11443357

[CIT0027] Lage, J. -L. D., Da Lage, J. -L., Binder, M., Hua-Van, A., Janeček, S., & Casane, D. (2013). Gene make-up: Rapid and massive intron gains after horizontal transfer of a bacterial α-amylase gene to basidiomycetes. BMC Evolutionary Biology, 13(1), 40. 10.1186/1471-2148-13-40 23405862 PMC3584928

[CIT0028] Lamarca, A. P., Mello, B., & Schrago, C. G. (2022). The performance of outgroup-free rooting under evolutionary radiations. Molecular Phylogenetics and Evolution, 169, 107434. 10.1016/j.ympev.2022.107434 35143961

[CIT0029] Li, M., Zhao, J., Tang, N., Sun, H., & Huang, J. (2018). Horizontal gene transfer from bacteria and plants to the *Arbuscular mycorrhizal* fungus. Frontiers in Plant Science, 9, 701. 10.3389/fpls.2018.00701 29887874 PMC5982333

[CIT0030] Libeskind-Hadas, R., Wu, Y. -C., Bansal, M. S., & Kellis, M. (2014). Pareto-optimal phylogenetic tree reconciliation. Bioinformatics, 30(12), i87–i95. 10.1093/bioinformatics/btu289 24932009 PMC4058917

[CIT0031] Linard, B., Ebersberger, I., McGlynn, S. E., Glover, N., Mochizuki, T., Patricio, M., Lecompte, O., Nevers, Y., Thomas, P. D., Gabaldón, T., Sonnhammer, E., Dessimoz, C., Uchiyama, I., & Consortium, Q. F. O. (2021). Ten years of collaborative progress in the quest for orthologs. Molecular Biology and Evolution, 38(8), 3033–3045. 33822172 10.1093/molbev/msab098 PMC8321534

[CIT0032] Manni, M., Berkeley, M. R., Seppey, M., Simão, F. A., & Zdobnov, E. M. (2021). BUSCO update: Novel and streamlined workflows along with broader and deeper phylogenetic coverage for scoring of eukaryotic, prokaryotic, and viral genomes. Molecular Biology and Evolution, 38(10), 4647–4654. 10.1093/molbev/msab199 34320186 PMC8476166

[CIT0033] Marcet-Houben, M., & Gabaldón, T. (2010). Acquisition of prokaryotic genes by fungal genomes. Trends in Genetics: TIG, 26(1), 5–8. 10.1016/j.tig.2009.11.007 19969385

[CIT0034] McDaniel, L. D., Young, E., Delaney, J., Ruhnau, F., Ritchie, K. B., & Paul, J. H. (2010). High frequency of horizontal gene transfer in the oceans. Science, 330(6000), 50. 10.1126/science.1192243 20929803

[CIT0035] Murphy, C. L., Youssef, N. H., Hanafy, R. A., Couger, M. B., Stajich, J. E., Wang, Y., Baker, K., Dagar, S. S., Griffith, G. W., Farag, I. F., Callaghan, T. M., & Elshahed, M. S. (2019). Horizontal gene transfer as an indispensable driver for evolution of *Neocallimastigomycota* into a distinct gut-dwelling fungal lineage. Applied and Environmental Microbiology, 85(15), e00988–e00919. 10.1128/AEM.00988-19 31126947 PMC6643240

[CIT0036] Naito, M., Morton, J. B., & Pawlowska, T. E. (2015). Minimal genomes of mycoplasma-related endobacteria are plastic and contain host-derived genes for sustained life within *Glomeromycota*. Proceedings of the National Academy of Sciences of the United States of America, 112(25), 7791–7796. 10.1073/pnas.1501676112 25964324 PMC4485128

[CIT0037] Naranjo-Ortiz, M. A., & Gabaldón, T. (2020). Fungal evolution: Cellular, genomic and metabolic complexity. Biological Reviews of the Cambridge Philosophical Society, 95(5), 1198–1232. 10.1111/brv.12605 32301582 PMC7539958

[CIT0038] NCBI Resource Coordinators. (2017). Database resources of the National Center for Biotechnology Information. Nucleic Acids Research, 45(D1), D12–D17. 27899561 10.1093/nar/gkw1071 PMC5210554

[CIT0039] Nee, S., May, R. M., & Harvey, P. H. (1994). The reconstructed evolutionary process. Philosophical Transactions of the Royal Society of London, Series B: Biological Sciences, 344(1309), 305–311. 10.1098/rstb.1994.0068 7938201

[CIT0040] Page, R. D., & Charleston, M. A. (1997). From gene to organismal phylogeny: Reconciled trees and the gene tree/species tree problem. Molecular Phylogenetics and Evolution, 7(2), 231–240. 10.1006/mpev.1996.0390 9126565

[CIT0041] Pawlowska, T. E., Gaspar, M. L., Lastovetsky, O. A., Mondo, S. J., Real-Ramirez, I., Shakya, E., & Bonfante, P. (2018). Biology of fungi and their bacterial endosymbionts. Annual Review of Phytopathology, 56, 289–309. 10.1146/annurev-phyto-080417-045914 30149793

[CIT0042] Persson, E., Kaduk, M., Forslund, S. K., & Sonnhammer, E. L. L. (2019). Domainoid: domain-oriented orthology inference. BMC Bioinformatics, 20(1), 523. 10.1186/s12859-019-3137-2 31660857 PMC6816169

[CIT0043] Poptsova, M. S., & Gogarten, J. P. (2010). Using comparative genome analysis to identify problems in annotated microbial genomes. Microbiology (Reading, England), 156(Pt 7), 1909–1917. 10.1099/mic.0.033811-0 20430813

[CIT0044] Rancurel, C., Legrand, L., & Danchin, E. G. J. (2017). Alienness: Rapid detection of candidate horizontal gene transfers across the tree of life. Genes, 8(10), 248. 10.3390/genes8100248 28961181 PMC5664098

[CIT0045] Richards, T. A., Leonard, G., Soanes, D. M., & Talbot, N. J. (2011). Gene transfer into the fungi. Fungal Biology Reviews, 25(2), 98–110. 10.1016/j.fbr.2011.04.003

[CIT0046] Richards, T. A., Soanes, D. M., Foster, P. G., Leonard, G., Thornton, C. R., & Talbot, N. J. (2009). Phylogenomic analysis demonstrates a pattern of rare and ancient horizontal gene transfer between plants and fungi. The Plant Cell, 21(7), 1897–1911. 10.1105/tpc.109.065805 19584142 PMC2729602

[CIT0047] Robinson, A. J., House, G. L., Morales, D. P., Kelliher, J. M., Gallegos-Graves, L. V., LeBrun, E. S., Davenport, K. W., Palmieri, F., Lohberger, A., Bregnard, D., Estoppey, A., Buffi, M., Paul, C., Junier, T., Hervé, V., Cailleau, G., Lupini, S., Nguyen, H. N., Zheng, A. O., … Chain, P. S. G. (2021). Widespread bacterial diversity within the bacteriome of fungi. Communications Biology, 4(1), 1168. 34621007 10.1038/s42003-021-02693-y PMC8497576

[CIT0048] Rodríguez-Ezpeleta, N., Brinkmann, H., Roure, B., Lartillot, N., Lang, B. F., & Philippe, H. (2007). Detecting and overcoming systematic errors in genome-scale phylogenies. Systematic Biology, 56(3), 389–399. 10.1080/10635150701397643 17520503

[CIT0049] Schönknecht, G., Weber, A. P. M., & Lercher, M. J. (2014). Horizontal gene acquisitions by eukaryotes as drivers of adaptive evolution. BioEssays: News and Reviews in Molecular, Cellular and Developmental Biology, 36(1), 9–20. 10.1002/bies.201300095 24323918

[CIT0050] Sjölander, K., Datta, R. S., Shen, Y., & Shoffner, G. M. (2011). Ortholog identification in the presence of domain architecture rearrangement. Briefings in Bioinformatics, 12(5), 413–422. 10.1093/bib/bbr036 21712343 PMC3178056

[CIT0051] Soliman, S. S. M., Baldin, C., Gu, Y., Singh, S., Gebremariam, T., Swidergall, M., Alqarihi, A., Youssef, E. G., Alkhazraji, S., Pikoulas, A., Perske, C., Venkataramani, V., Rich, A., Bruno, V. M., Hotopp, J. D., Mantis, N. J., Edwards, J. E., Filler, S. G., Chamilos, G., … Ibrahim, A. S. (2021). Mucoricin is a ricin-like toxin that is critical for the pathogenesis of mucormycosis. Nature Microbiology, 6(3), 313–326. 10.1038/s41564-020-00837-0 PMC7914224 33462434

[CIT0052] Steel, M., & McKenzie, A. (2001). Properties of phylogenetic trees generated by Yule-type speciation models. Mathematical Biosciences, 170(1), 91–112. 10.1016/s0025-5564(00)00061-4 11259805

[CIT0053] Tabima, J. F., Trautman, I. A., Chang, Y., Wang, Y., Mondo, S., Kuo, A., Salamov, A., Grigoriev, I. V., Stajich, J. E., & Spatafora, J. W. (2020). Phylogenomic analyses of non-Dikarya fungi supports horizontal gene transfer driving diversification of secondary metabolism in the amphibian gastrointestinal symbiont. G3, 10(9), 3417–3433. 32727924 10.1534/g3.120.401516 PMC7466969

[CIT0054] Tan, G., Muffato, M., Ledergerber, C., Herrero, J., Goldman, N., Gil, M., & Dessimoz, C. (2015). Current methods for automated filtering of multiple sequence alignments frequently worsen single-gene phylogenetic inference. Systematic Biology, 64(5), 778–791. 10.1093/sysbio/syv033 26031838 PMC4538881

[CIT0055] Udny Yule, G. (1925). A mathematical theory of evolution based on the conclusions of Dr. J. C. Willis, F.R.S. Journal of the Royal Statistical Society, 88(3), 433. 10.2307/2341419

[CIT0056] Uehling, J., Deveau, A., & Paoletti, M. (2017). Do fungi have an innate immune response? An NLR-based comparison to plant and animal immune systems. PLoS Pathogens, 13(10), e1006578. 10.1371/journal.ppat.1006578 29073287 PMC5658179

[CIT0057] Uehling, J., Gryganskyi, A., Hameed, K., Tschaplinski, T., Misztal, P. K., Wu, S., Desirò, A., Vande Pol, N., Du, Z., Zienkiewicz, A., Zienkiewicz, K., Morin, E., Tisserant, E., Splivallo, R., Hainaut, M., Henrissat, B., Ohm, R., Kuo, A., Yan, J., … Bonito, G. (2017). Comparative genomics of *Mortierella elongata* and its bacterial endosymbiont *Mycoavidus cysteinexigens*. Environmental Microbiology, 19(8), 2964–2983. 10.1111/1462-2920.13669 28076891

[CIT0058] Van Etten, J., & Bhattacharya, D. (2020). Horizontal gene transfer in eukaryotes: Not if, but how much? Trends in Genetics, 36(12), 915–925. 10.1016/j.tig.2020.08.006 33012528

[CIT0059] Venice, F., Desirò, A., Silva, G., Salvioli, A., & Bonfante, P. (2020). The mosaic architecture of NRPS-PKS in the *Arbuscular mycorrhizal* fungus *Gigaspora margarita* shows a domain with bacterial signature. Frontiers in Microbiology, 11, 581313. 10.3389/fmicb.2020.581313 33329443 PMC7732545

[CIT0060] Venice, F., Ghignone, S., Salvioli di Fossalunga, A., Amselem, J., Novero, M., Xianan, X., Sędzielewska Toro, K., Morin, E., Lipzen, A., Grigoriev, I. V., Henrissat, B., Martin, F. M., & Bonfante, P. (2020). At the nexus of three kingdoms: The genome of the mycorrhizal fungus *Gigaspora margarita* provides insights into plant, endobacterial and fungal interactions. Environmental Microbiology, 22(1), 122–141. 10.1111/1462-2920.14827 31621176

[CIT0061] Virtanen, P., Gommers, R., Oliphant, T. E., Haberland, M., Reddy, T., Cournapeau, D., Burovski, E., Peterson, P., Weckesser, W., Bright, J., van der Walt, S. J., Brett, M., Wilson, J., Jarrod Millman, K., Mayorov, N., Nelson, A. R. J., Jones, E., Kern, R., & Larson, E.; SciPy 1. 0 Contributors. (2020). SciPy 1.0: Fundamental algorithms for scientific computing in Python. Nature Methods, 17(3), 261–272. 10.1038/s41592-019-0686-2 32015543 PMC7056644

[CIT0062] Voigt, K., James, T. Y., Kirk, P. M., Santiago, A. L. C. M. de A., Waldman, B., Griffith, G. W., Fu, M., Radek, R., Strassert, J. F. H., Wurzbacher, C., Jerônimo, G. H., Simmons, D. R., Seto, K., Gentekaki, E., Hurdeal, V. G., Hyde, K. D., Nguyen, T. T. T., & Lee, H. B. (2021). Early-diverging fungal phyla: Taxonomy, species concept, ecology, distribution, anthropogenic impact, and novel phylogenetic proposals. Fungal Diversity, 109(1), 59–98. 10.1007/s13225-021-00480-y 34608378 PMC8480134

[CIT0063] Wade, T., Rangel, L. T., Kundu, S., Fournier, G. P., & Bansal, M. S. (2020). Assessing the accuracy of phylogenetic rooting methods on prokaryotic gene families. PLoS One, 15(5), e0232950. 10.1371/journal.pone.0232950 32413061 PMC7228096

[CIT0064] Wisecaver, J. H., Slot, J. C., & Rokas, A. (2014). The evolution of fungal metabolic pathways. PLoS Genetics, 10(12), e1004816. 10.1371/journal.pgen.1004816 25474404 PMC4256263

[CIT0065] Wootton, J. C., & Federhen, S. (1996). Analysis of compositionally biased regions in sequence databases. Methods in Enzymology, 266, 554–571. 10.1016/s0076-6879(96)66035-2 8743706

[CIT0066] Wurlitzer, J. M., Stanišić, A., Wasmuth, I., Jungmann, S., Fischer, D., Kries, H., & Gressler, M. (2021). Bacterial-like nonribosomal peptide synthetases produce cyclopeptides in the Zygomycetous fungus *Mortierella alpina*. Applied and Environmental Microbiology, 87(3), e02051–e02020. 10.1128/AEM.02051-20 33158886 PMC7848919

[CIT0067] Yanai, I., Wolf, Y. I., & Koonin, E. V. (2002). Evolution of gene fusions: Horizontal transfer versus independent events. Genome Biology, 3(5), research0024. 10.1186/gb-2002-3-5-research0024 12049665 PMC115226

