# Supplementary material for: The interkingdom horizontal gene transfer in 44 early diverging fungi boosted their metabolic, adaptive, and immune capabilities
Source: Evol Lett. 2024 Mar 5;8(4):526–38. doi: 10.1093/evlett/qrae009 (PMC11291939; doi:10.1093/evlett/qrae009)
Supplement: qrae009_suppl_Supplementary_Figures [file qrae009_suppl_supplementary_figures.pdf]

The interkingdom horizontal gene transfer in 44 early diverging fungi boosted their metabolic, adaptive and immune capabilities

Michał Aleksander Ciach*[1,3], Julia Pawłowska[2], Paweł Górecki[1] and Anna Muszewska*[3]

[1] Faculty of Mathematics, Informatics and Mechanics, University of Warsaw, Stefana Banacha 2, 02-097 Warsaw, Poland

[2] Institute of Evolutionary Biology, Faculty of Biology, Biological and Chemical Research Centre, University of Warsaw, ul. Żwirki i Wigury 101, 02-089 Warsaw, Poland;

[3] Institute of Biochemistry and Biophysics, Polish Academy of Sciences, Pawinskiego 5A, 02-106 Warsaw, Poland

*corresponding authors:

Michał Ciach: m.ciach@mim.uw.edu.pl

Anna Muszewska: musze@ibb.waw.pl

Table of content

Supplementary Figures 3

Supplementary Fig. S1. Left: the numbers of phylogenetic trees with different sequence substitution models fitted by ModelFinder of iqTree. Right: Causes of an incomplete support for the HGT hypothesis in fungal subtrees (in incongruent locations) discarded from the statistical analysis of HGT properties. **3**

Supplementary Fig. S2 In the protein clusters with predominantly non-fungal taxonomic composition, the number of incongruent fungal clades is highly correlated with the number of congruent fungal clades (ρ=0.80, p<1e-12). Incongruent (displaced) clades are assumed to be a stronger indication of HGT than congruent (non-displaced) clades, as the latter may potentially also be caused by sequence homoplasy and simple vertical evolution. A high correlation of the numbers of both types of clades across the species tree of 44 EDF suggests that HGT is a more likely explanation for the congruent clades, as other evolutionary mechanisms would be expected to produce a more uniform distribution over the tree. **4**

Supplementary Fig. S3. Numbers of contaminants removed from the 44 EDF proteomes in both stages of contaminant filtering. The first stage discards contigs without any fungal homology, the second stage further discards contigs without any fungal first hit in 10 randomly sampled proteins. 5

Supplementary Fig. S4. The size of the proteome (blue) and the number of detected xenologs (orange) for 44 fungi analyzed in this study. Note the different axis scales for the blue and orange bars. 6

Supplementary Fig. S5. The dynamics of sequence length and low-complexity proportion of proteins after transfer. Left: The difference between the average lengths of the fungal acceptor and the donor group sequences. Right: The difference between the average proportions of sequences masked with ncbi-seg. 7

Supplementary Fig. S6. Additional results of the WolfPSort subcellular location prediction software. A) Location consistency of fungal xenologs in a xenologous family, defined as the fraction of fungal proteins with identical location in a protein cluster. B) The comparison of location prediction for proteome background and decoy proteins generated with random permutations of protein sequences. C) The distribution of WolfPSort prediction scores for the background proteins, decoy proteins, and xenologs. 8

Supplementary Fig. S7. The dependence of the number of introns in bacteria-derived fungal xenologs on the total average number of substitutions per site after transfer (measured as the sum of lengths of branches joining a given fungal xenolog with the transfer origin node, i.e. the gene tree's internal node to which the corresponding fungal subtree is attached). Dashed line fitted with linear regression without intercept. The estimated slope is equal to 0.6 introns per substitution per site. **9**

Supplementary Fig. S8. Detailed BlastP results and phylogenetic trees for the protein ORY15655.1, containing a xenologous region as well as a short region of fungal origin. The influence of the fungal signal is visible as a distortion of the topology of the tree constructed from the whole sequence compared to trees constructed from different regions. The tree constructed from the N-terminal region of 530 aa supports a bacterial origin of the sequence, the tree constructed from the C-terminal region of 180 aa supports a fungal origin of the sequence, while the tree constructed from the full sequence shows a mixed taxonomy. All trees were constructed from the first 500 BLASTP hits of respective query sequence using the BLAST web suite (full sequence; residues 1:530; residues 531:710). 10

Supplementary Fig. S9. A multiple sequence alignment of the R. globosum ORY15655.1 with its four post-transfer paralogs. The ORY15655.1 is an intron-rich bacteria-derived xenolog with a bacterial Glycoside_hydrolase_SF domain and a post-transfer fused fungal C-terminal domain Mg_trans_NIPA. On the other hand, the protein has lost an N-terminal bacteria-derived fragment visible in ORY15656.1 and ORY29292.1. This indicates a complex evolution of the gene after incorporation in the fungal genome, involving gene duplication, domain gain and acquisition in the paralogs, and intronization. **11**

Supplementary Data **12**

Supplementary Results **12**

Supplementary Methods 13

A) HGT identification pipeline 13

B) Avoiding false positives in high-throughput HGT studies 22

C) Controlling long branches in gene trees 28

D) Detecting horizontal gene transfer in unrooted gene trees 30

References 37

Supplementary Figures

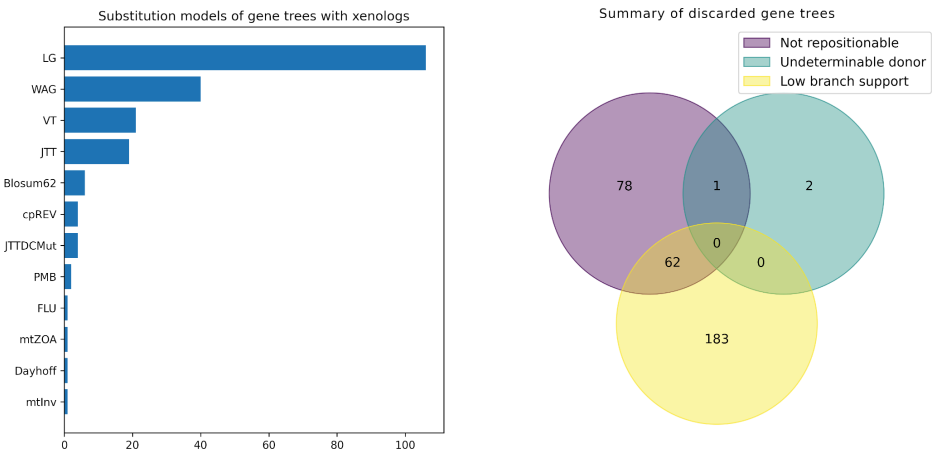

Supplementary Fig. S1. Left: the numbers of phylogenetic trees with different sequence substitution models fitted by ModelFinder of iqTree. Right: Causes of an incomplete support for the HGT hypothesis in fungal subtrees (in incongruent locations) discarded from the statistical analysis of HGT properties.

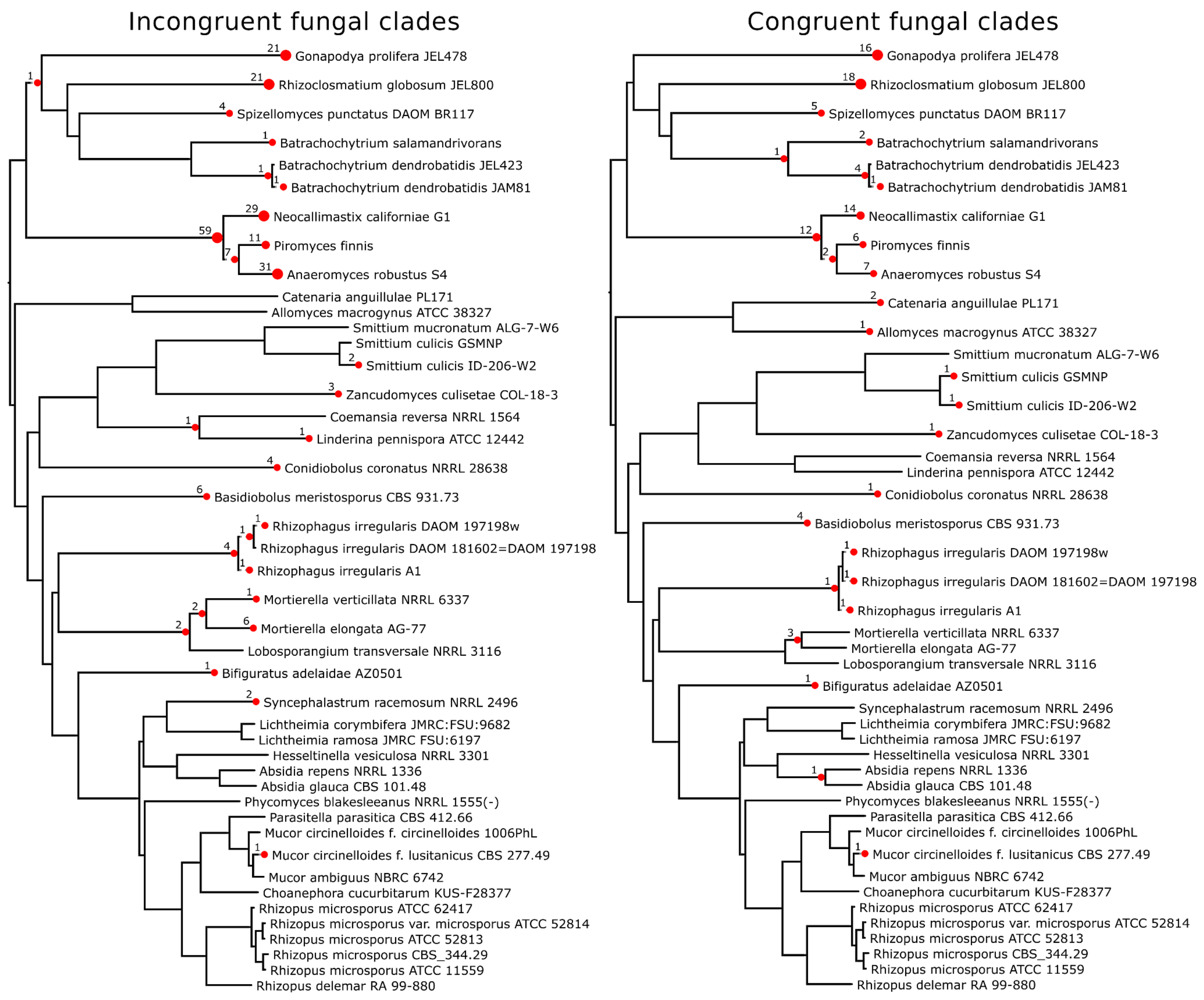

Supplementary Fig. S2 In the protein clusters with predominantly non-fungal taxonomic composition, the number of incongruent fungal clades is highly correlated with the number of congruent fungal clades (ρ=0.80, p<1e-12). Incongruent (displaced) clades are assumed to be a stronger indication of HGT than congruent (non-displaced) clades, as the latter may potentially also be caused by sequence homoplasy and simple vertical evolution. A high correlation of the numbers of both types of clades across the species tree of 44 EDF suggests that HGT is a more likely explanation for the congruent clades, as other evolutionary mechanisms would be expected to produce a more uniform distribution over the tree.

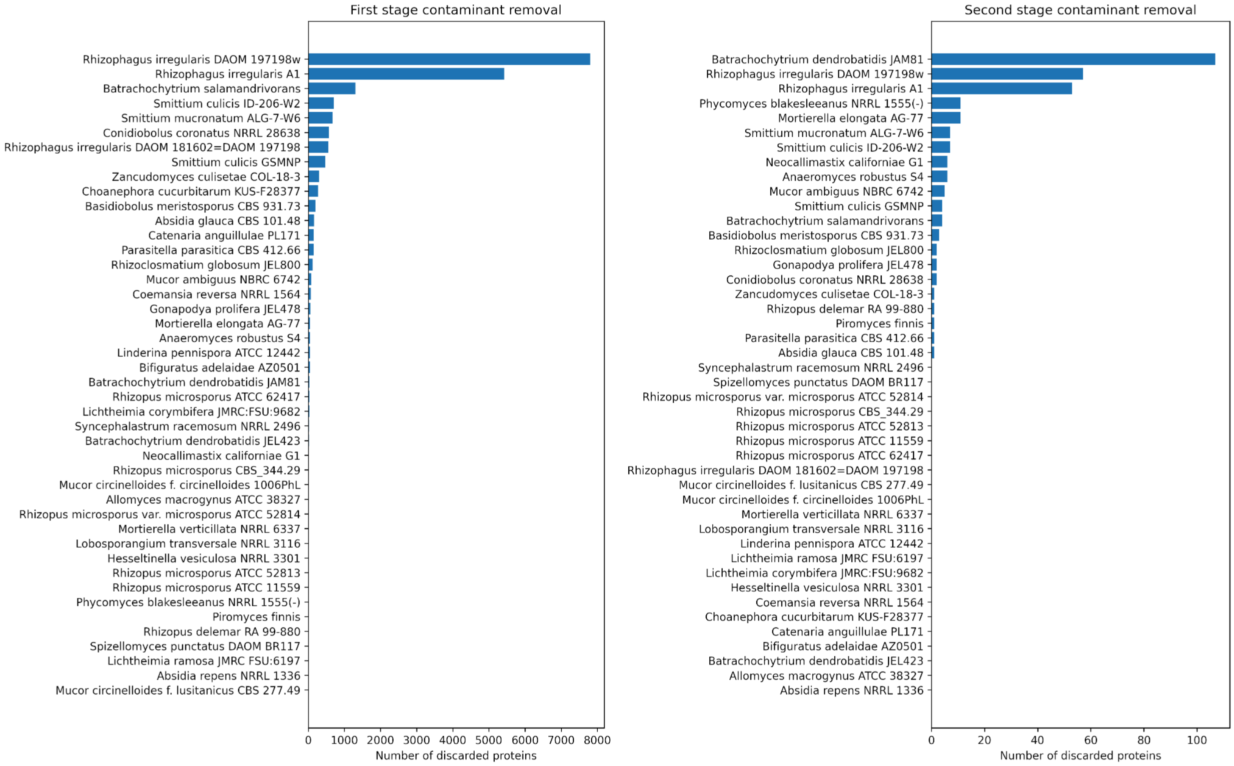

Supplementary Fig. S3. Numbers of contaminants removed from the 44 EDF proteomes in both stages of contaminant filtering. The first stage discards contigs without any fungal homology, the second stage further discards contigs without any fungal first hit in 10 randomly sampled proteins.

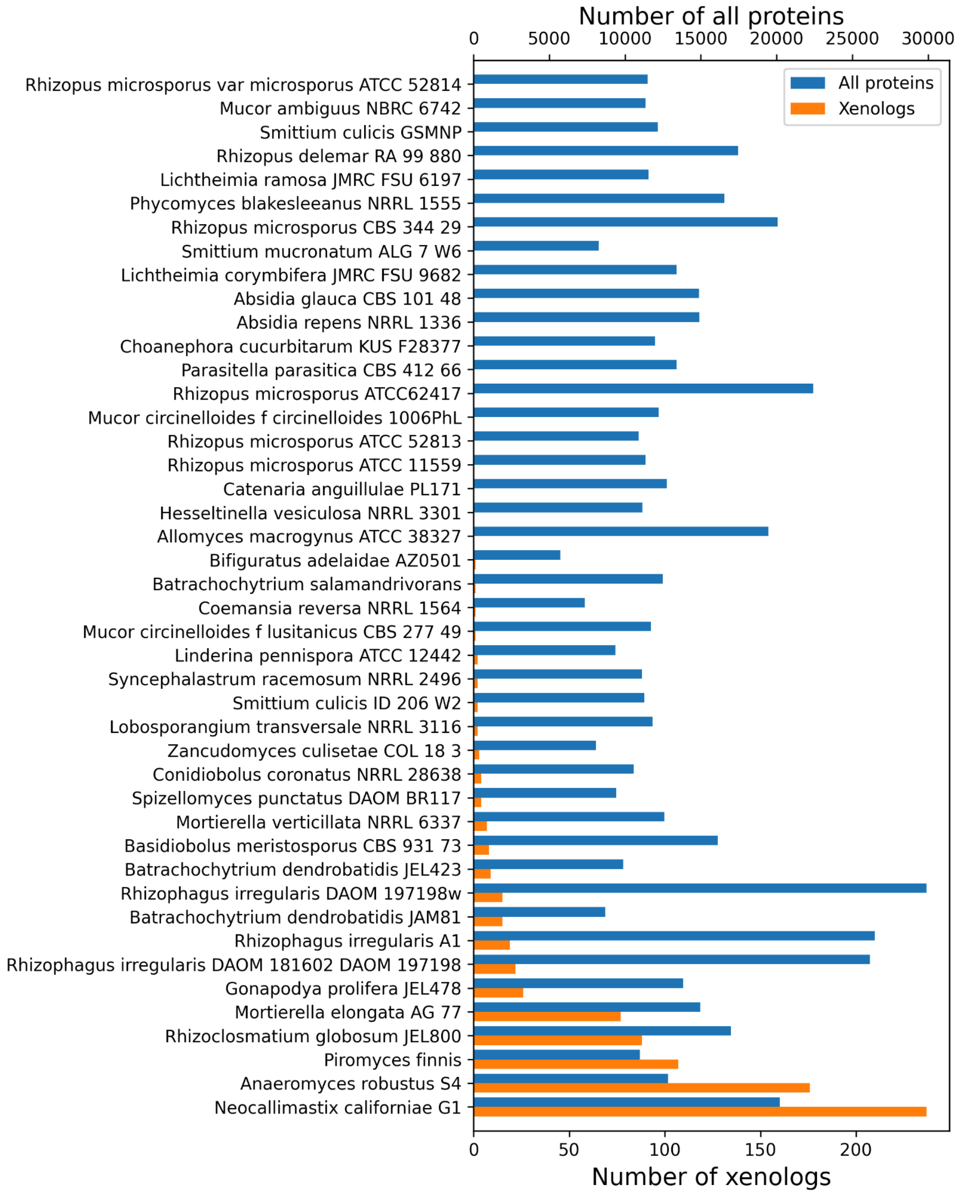

Supplementary Fig. S4. The size of the proteome (blue) and the number of detected xenologs (orange) for 44 fungi analyzed in this study. Note the different axis scales for the blue and orange bars.

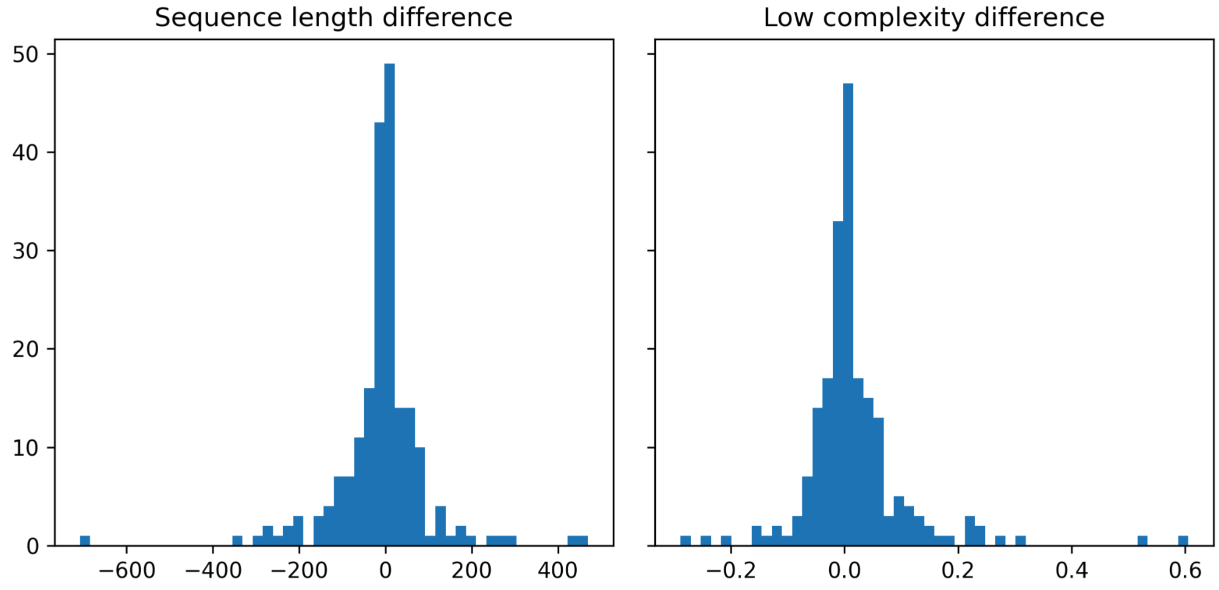

Supplementary Fig. S5. The dynamics of sequence length and low-complexity proportion of proteins after transfer. Left: The difference between the average lengths of the fungal acceptor and the donor group sequences. Right: The difference between the average proportions of sequences masked with ncbi-seg.

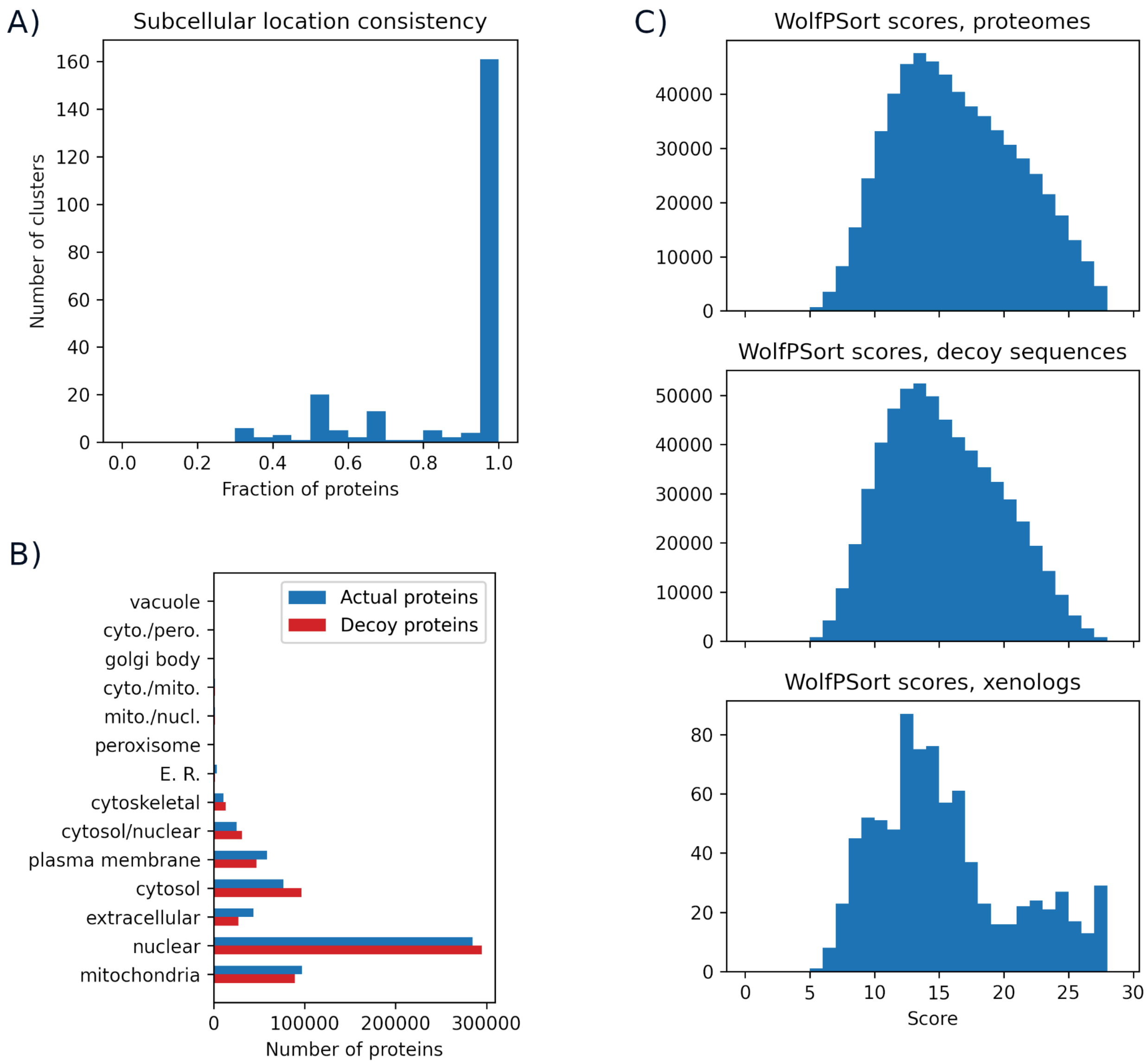

Supplementary Fig. S6. Additional results of the WolfPSort subcellular location prediction software. A) Location consistency of fungal xenologs in a xenologous family, defined as the fraction of fungal proteins with identical location in a protein cluster. B) The comparison of location prediction for proteome background and decoy proteins generated with random permutations of protein sequences. C) The distribution of WolfPSort prediction scores for the background proteins, decoy proteins, and xenologs.

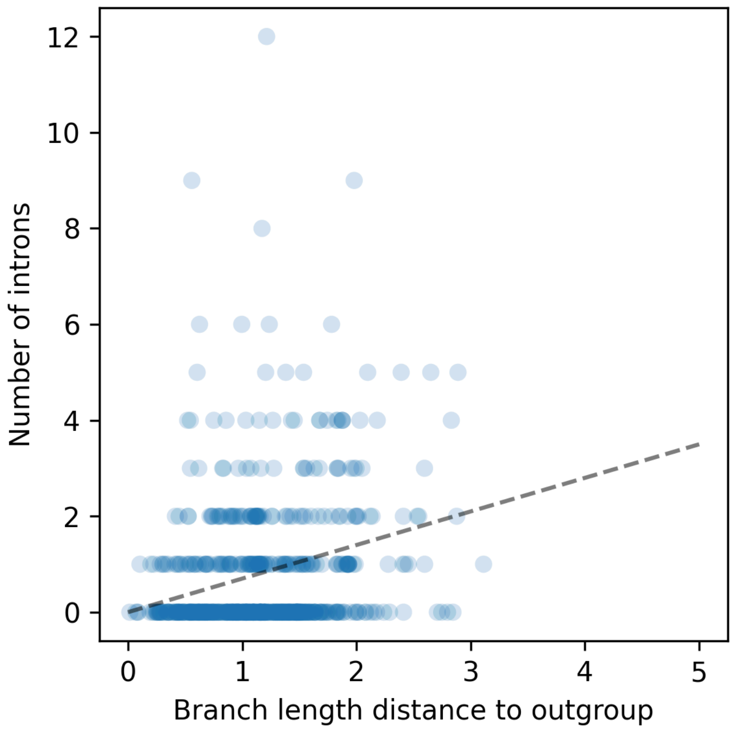

Supplementary Fig. S7. The dependence of the number of introns in bacteria-derived fungal xenologs on the total average number of substitutions per site after transfer (measured as the sum of lengths of branches joining a given fungal xenolog with the transfer origin node, i.e. the gene tree's internal node to which the corresponding fungal subtree is attached). Dashed line fitted with linear regression without intercept. The estimated slope is equal to 0.6 introns per substitution per site.

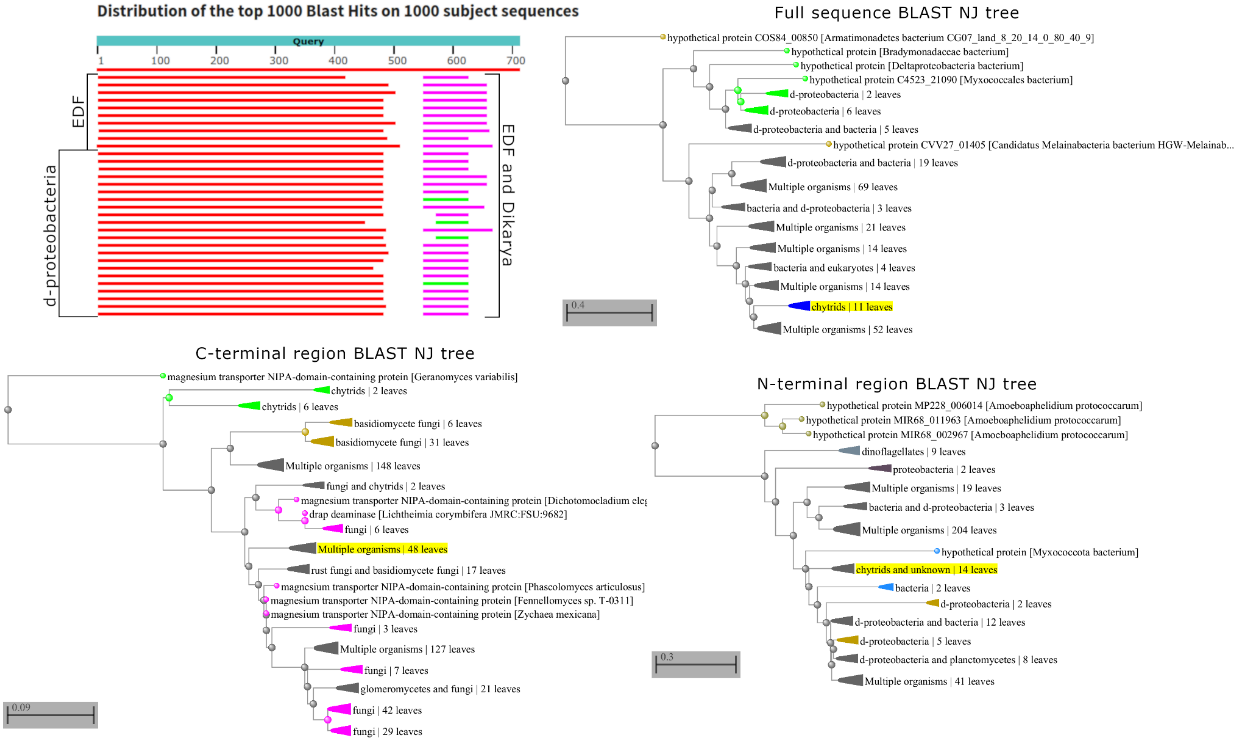

Supplementary Fig. S8. Detailed BlastP results and phylogenetic trees for the protein ORY15655.1, containing a xenologous region as well as a short region of fungal origin. The influence of the fungal signal is visible as a distortion of the topology of the tree constructed from the whole sequence compared to trees constructed from different regions. The tree constructed from the N-terminal region of 530 aa supports a bacterial origin of the sequence, the tree constructed from the C-terminal region of 180 aa supports a fungal origin of the sequence, while the tree constructed from the full sequence shows a mixed taxonomy. All trees were constructed from the first 500 BLASTP hits of respective query sequence using the BLAST web suite (full sequence; residues 1:530; residues 531:710).

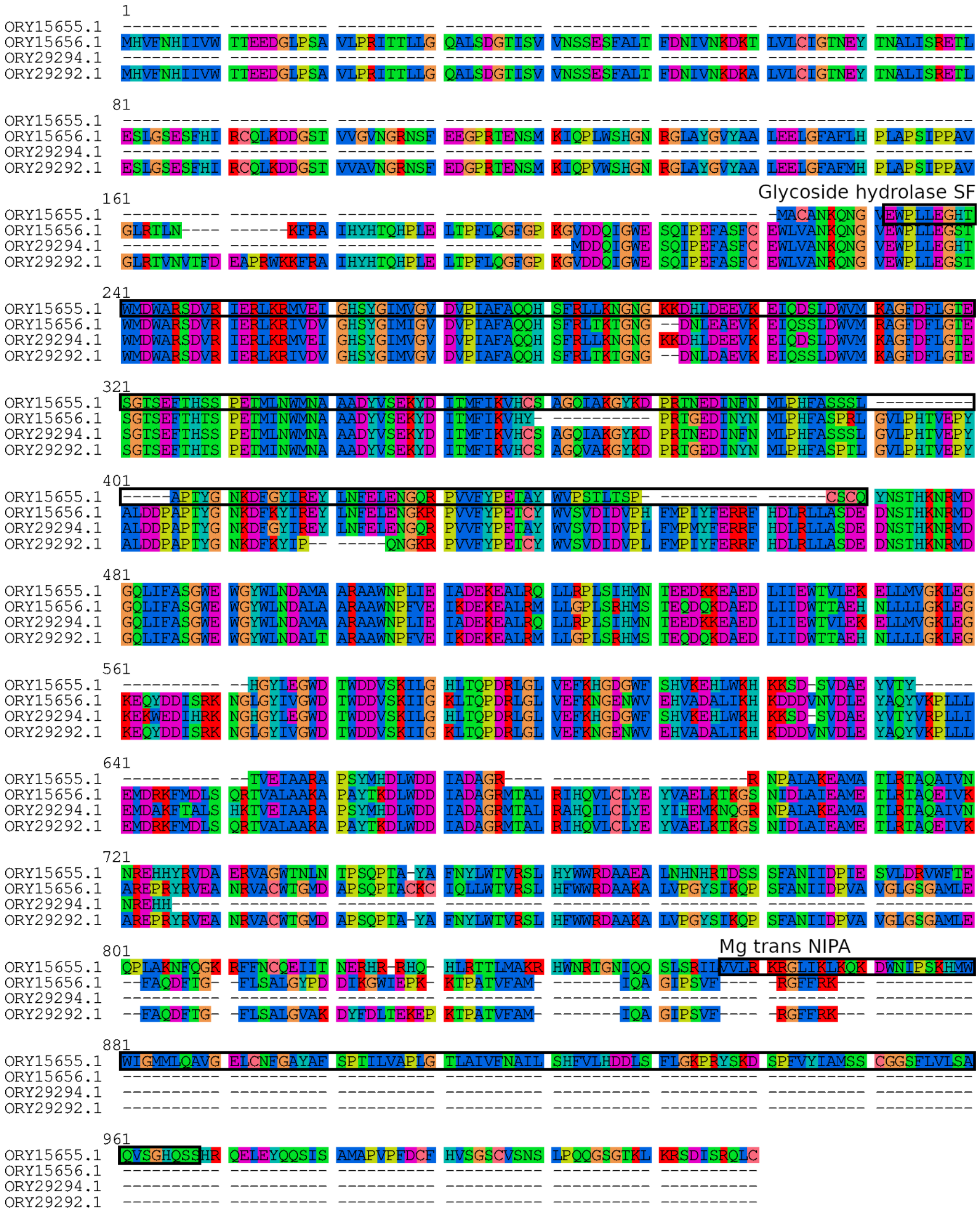

Supplementary Fig. S9. A multiple sequence alignment of the *R. globosum* ORY15655.1 with its four post-transfer paralogs. The ORY15655.1 is an intron-rich bacteria-derived xenolog with a bacterial Glycoside_hydrolase_SF domain and a post-transfer fused fungal C-terminal domain Mg_trans_NIPA. On the other hand, the protein has lost an N-terminal bacteria-derived fragment visible in ORY15656.1 and ORY29292.1. This indicates a complex evolution of the gene after incorporation in the fungal genome, involving gene duplication, domain gain and acquisition in the paralogs, and intronization.

Supplementary Data

Supplementary Table S1 is an xls file with multiple sheets:

Protein summary - a list of well-supported xenologs

Organism summary - number of xenologs in different proteomes

Domain summary - occurence of protein domains in clusters

Cluster summary - all identified protein clusters containing fungal xenologs, including unsupported ones

Assemblies - list of assemblies analysed

Supplementary Data - Phylogenetic trees of supported and unsupported HGT along the HGT detection pipeline are deposited under https://doi.org/10.5281/zenodo.10649525

Supplementary Results

Filtering of data

The first step removed 19578 proteins (**Supplementary Fig. S3**). This constitutes 3% of the 44 proteomes jointly, and 2% of each proteome on average. The number of removed proteins varied highly between proteomes, from 1 in *Absidia repens* NRRL 1336 and *Spizellomyces punctatus* DAOM BR117 through 203 in *Basidiobolus meristosporus* CBS 931.73 up to 5426 in *Rhizophagus irregularis* A1. The extensive contamination of the *R. irregularis* A1 proteome is in part likely caused by an associated *Lysinibacillus* bacterium.

The second, refined step removed additional 292 proteins. Here, the removed numbers varied to a lesser extent, with the largest being 107 for *Batrachochytrium dendrobatidis* JAM81; notably, the first stage discarded only 36 proteins in this fungus. This was followed by 57 and 53

proteins further removed from *Rhizophagus irregularis* DAOM 197198w and *Rhizophagus irregularis* A1.

Horizontal transfer of trasposons (HTT)

We have detected a few transfers (n=7) of mobile elements, including a transfer of a PiggyBac element from *Phytophthora* oomycetes to *Smittium culicis* (OMJ27075.1) and of a retrotransposon from oomycete *Globisporangium splendens* to *Rhizoclosmatium globosum* (ORY50945.1). We have also identified several phage proteins without a clear function in fungi, eg. a phage tail lysozyme PF18013 in *Piromyces finnis* protein ORX49608.1 transferred from Clostridium bacteria. However, most of the potential transposon and viral xenologs were likely excluded from our data set because of the usage of protein sequences instead of genomic sequences for HGT inference and our filtering steps.

Xenolog localization predictions

Despite being lower than for the background, the percentage of xenologs with mitochondrial and nuclear predictions is sill unexpectedly high, and may suggest that WolfPsort is biased towards these locations. To verify this hypothesis, we have generated a decoy dataset by randomly permuting each sequence from the 44 proteomes. The predicted distribution of locations was very similar for the proteome background and the decoy dataset, with the nucleus as the most common location, and the decoy proteins attained similar prediction scores (**Supplementary Fig. S6**). This was likely caused by the fact that WolfPSort is based on the *k*-Nearest Neighbours classification algorithm, which tends to reflect the distribution of the training dataset, in this case, fungal proteomes. The prediction may be further complicated by the fact that xenolog sequences have a non-fungal origin.

Supplementary Methods

A) HGT identification pipeline

Our HGT identification approach is visualized in **Fig. SM1** (in the manuscript named **Fig. 1** and copied below). A tutorial-like description of the pipeline with all the necessary commands and scripts required to reproduce the results is available at https://github.com/mciach/HGTin44EDF. First, low-complexity regions in all target proteins were masked using ncbi-seg with relaxed settings (window length 12, trigger complexity 1.8, extension complexity 2.0) (John C. Wootton and Federhen 1993). Target proteins with less than 30 non-masked amino acids, more than 2000 characters, or more than 50% masking were discarded as prone to produce false-positive hits (step (1) in Fig. SM1). This is because long proteins often have a complex multi domain structure, with domain shuffling and dynamic domain loss/gain, which impacts both alignment quality and phylogeny inference (Stolzer et al. 2015). This procedure removed 5949 out of 606201 proteins. Note that, although it increases the reliability of prediction, it also comes at a cost of missing some of the well documented transfers of long proteins, such as the transfer of NRPS to *Mortierella* species (Wurlitzer et al. 2021b). However, since less than 1% proteins are removed overall, it is unlikely to have a noticeable influence on the statistical patterns.

Target protein homology within the 44 target proteomes was evaluated using the blastp suite with an e-value threshold 1e-05 (NCBI Resource Coordinators 2017). Target proteins with homologs in fungi belonging to more than one taxonomic family were discarded as likely inherited vertically (step (2) in Fig. SM1). Contigs without any protein coding gene with detectable homology to the other 44 target fungal proteomes were discarded as likely genome contaminants (first-stage contaminant filtering; step (3) in Fig. SM1).

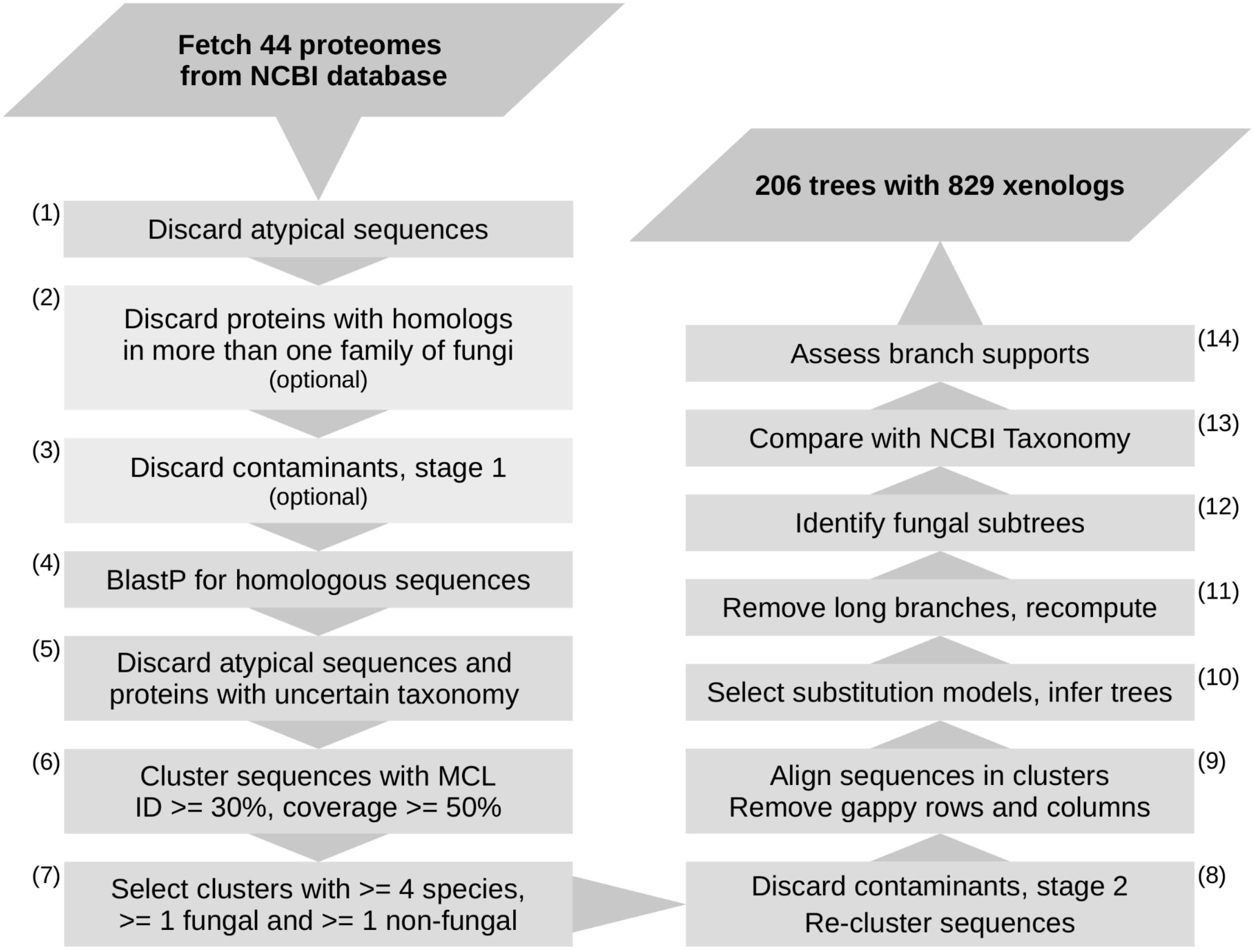

Fig. SM1. A flow chart of the HGT identification pipeline.

Sequences of proteins remaining after step (3) were blasted against a local copy of the Non-Redundant NCBI database (last updated December 01, 2020), using blastp suite with an e-value threshold 1e-04 and maximum of 500 target sequences. Detected homologs were masked using ncbi-seg (default settings). Proteins with less than 30 non-masked amino acids, more than 2000 characters, or more than 75% masking were discarded (step (5) in Fig. SM1). The taxonomic lineage of each homolog was inspected using the NCBI Taxonomy database. Only homologs from bacteria, archaea and eukarya were retained in order to discard viral and artificial sequences due to their uncertain location in the species tree (step (5) in Fig. SM1). Proteins with ’unclassified’, ‘uncultured’, ‘environmental’, or ‘incertae sedis’ NCBI Taxonomy

keywords anywhere in their lineage were discarded because their placement on the species tree is undefined (step (5) in Fig. SM1). An important exception here is that proteins from the 44 target fungal proteomes analyzed in this work were not subjected to this filtering step, because some of the 44 fungi belong to *incertae sedis* families, and therefore applying this filter would fully discard their proteomes.

All remaining proteins were clustered with MCL (step (6) in Fig. SM1). First, an all-vs-all BlastP was performed, with an increased word size to increase specificity and speed up computations (-word_size 6 -threshold 21 -evalue 1e-06). Only HSP matches with identity at least 30% and covering at least 50% of both the query and the subject proteins were retained. This step was essential to avoid cluster merging caused by long, multi-domain proteins and by small but highly conserved regions. During the development of our pipeline, we have inspected two approaches: either just 50% of the query, as in (Keeling et al., 2022), or 50% of both the query and the subject. We elaborate on why we decided to proceed with the latter approach in Supplementary Methods B. Note, however, that this requirement means that our method may be biased against multi-domain (and, in particular, eukaryotic) proteins and products of gene fusions.

The resulting protein homology graph was clustered with MCL (inflation parameter = 1.7). The results were filtered by discarding clusters without target proteins, with less than 4 different species (the minimum number of species required to get an unrooted phylogenetic tree), with species composition being more than 60% fungal, and clusters with fungal sequences from more than a single phylum. These clusters were assumed to be either inherited vertically or exhibiting evolutionary patterns too complex for an automated analysis (step (7) in Fig. SM1).

For each target protein remaining after step (7), we have inspected the contig of the protein's coding gene in order to discard potentially contaminating sequences (step (8) of Fig. SM1). This

step can be just as well performed at any previous stage of the analysis; however, performing it after the initial filtering of sequence clusters greatly decreases its computational cost. For each contig containing a gene encoding a target protein, 10 proteins were selected at random (or, if the contig contained less than 10 proteins, all of its proteins other than the target sequence). The selected proteins were blasted against the NR database. Contigs with no detected protein coding region with a fungal first blast hit were discarded as likely contaminants. Note that in this step, we retain a contig even if we detect only one fungal protein and nine non-fungal (e.g. bacterial) ones. We have analyzed different thresholds when designing the pipeline, and while the threshold of 1 fungal protein removes 292 sequences (out of 3387), increasing it to values between 2 and 5 removes additional 90, 120, 145, and 175 sequences. Therefore, the impact of setting the threshold to 1 is larger than the impact of increasing it. As a consequence, the threshold of 1 fungal protein seems the most natural.

The proteins remaining after this filtering step were clustered again with MCL using the same strategy. The re-clustering was performed because removing large numbers of nodes from a homology graph can alter the structure of clusters. The clusters resulting from step (8) were aligned using mafft 3.7 (Nakamura et al. 2018) with E-INS-i strategy, recommended for the alignment of general sequences due to having fewer assumptions than other strategies (--genafpair --maxiterate 5000). Alignments were then filtered by discarding constant columns and columns with more than 80% gaps, and next by discarding sequences with less than 20 non-gap characters or with more than 75% gaps (step (9) in Fig. SM1).

Maximum likelihood (ML) trees were inferred using IqTree (Minh et al. 2020) with automated model selection (step (10) in Fig. SM1). Branch supports were assessed with UFBoot, 2000 replicates. Leaves were discarded if no fungal leaf was closer than 3.33 substitutions per site (corresponding to an average of 15% sequence identity (Grishin 1995)). This step was

performed in order to reduce the complexity of the trees and focus on the neighborhoods of fungal sequences. Next, long branches (both terminal and internal ones) were removed iteratively until all branches were no longer than 1.598 substitutions per site (corresponding to an average of 30% sequence identity; step (11) in Fig. SM1; see the Supplementary Methods C for a detailed description of the branch-cutting algorithm). Resulting trees were retained if they contained proteins from at least one target fungal species and three non-fungal species (the minimal set of species required to be able to infer HGT using phylogenetics). This step was performed in order to limit the influence of long branches on the tree topology and remove outlying (not evolutionarily related) sequences from the clusters. Namely, in Maximum Likelihood phylogenetics, long branches join highly dissimilar sequences (inferred ancestral sequences in the case of internal nodes). This suggests that the corresponding cluster consists of two sets of distantly related sequences, and we consider such trees unreliable. We have decided that instead of discarding such trees, we can analyze a suitable sub-tree focused on the target fungi. The splitting of internal branches is a simple way of selecting those sub-trees.

While designing our pipeline, we have analyzed how often our procedure cuts internal and leaf branches, and the impact of this step on the resulting number of gene trees and the distribution of their sizes. There were two common scenarios where our method cut the branches: either individual fungal leaves with long branches, or large and complex trees. In total, the procedure removed 190 branches, 160 of which were long leaves and only 30 were internal branches. The procedure had a noticeable, but a rather small influence on the number of trees: by cutting 689 trees (and removing the resulting parts which don't contain fungi), we have obtained 586 resulting trees. The 103 trees were lost because they contained single fungal proteins attached with long branches. On the other hand, the procedure has decreased the largest tree size from 1249 to 850 leaves. The fact that the procedure targets long leaves (whose placement on the tree is inherently unreliable even if they're well-supported) or large and complex trees (which

are inherently difficult to infer accurately) is desirable.

For all trees remaining after step (11), their sequences were re-aligned and the alignments were filtered using the same strategy. Next, ML trees were recomputed using the same strategy. This step was performed because removing sequences can alter the topologies, branch lengths, and branch supports of ML trees.

For each gene tree, a corresponding species tree was downloaded from a local copy of the NCBI Taxonomy database. For each gene tree/species tree pair, all fungal subtrees were identified (step (12) in **Fig. SM1**). Next, the species composition of the neighboring non-fungal subtrees was compared between the trees (step (13) in **Fig. SM1**; see Fig. 2 for a visual description and the Supplementary Material for a detailed description of this step). Fungal subtrees which had different locations in the gene tree and the species tree were considered displaced and therefore indicative of HGT. Otherwise, if the location of a fungal subtree agreed in both trees, it was deemed as a possible vertical inheritance or sequence homoplasy and therefore not sufficient evidence to infer a horizontal gene transfer. In the case of polytomous species trees, we have inspected all their possible binarizations and inferred HGT only if it was supported by all binarizations.

To further limit the number of false positive results, we discarded gene trees in which fungal subtrees could not be repositioned to their correct locations, i.e. gene trees with no branch giving the same location (measured in terms of species composition of neighboring subtrees) as in the species tree (see **Fig. SM2**). In gene trees without such a branch, any location of the fungal subtree would be artifactually indicative of an HGT. This approach allows for other transfers and evolutionary events within the neighboring subtrees, but discards trees with transfers between the neighboring subtrees or gene tree inference errors as too complex for an automated analysis and prone to return false positive results.

For each fungal subtree, an average of its UFBoot support value and the support of the two neighboring subtrees was computed (step (14) in **Fig. SM1;** see also **Fig. SM2**). Candidate HGT fungal subtrees for which the average support of the three branches was lower than 90% were discarded as weakly supported HGTs. We have used a threshold of 90% rather than the recommended 95% for the following reasons. The UFBoot support for a given is an unbiased estimate of the probability that the branch exists in the true tree, provided that the substitution model is correctly specified (Hoang et al., 2018). Therefore, a branch with 90% probability can still be considered well-supported, especially for statistical studies. In our setting, higher support values are particularly difficult to attain, since our trees are inherently difficult to infer due to a high-throughput setting and complex sequence evolution associated with HGT. Furthermore, setting a support requirement for three branches is a more stringent constraint than setting a threshold for a single branch, and a slight decrease in the threshold allows us to balance it out in order not to lose too many cases of HGT. On the other hand, requiring that all the three branches are well-supported is necessary to determine the gene donor.

For the remaining subtrees, we have estimated the origin of the transfer by inspecting the last common ancestors (LCAs) of species in its two neighboring subtrees and the joint LCA of both neighboring subtrees, ignoring fungal species. If one of the neighbors had the same LCA as the whole neighborhood, we assumed that the other neighbor was the putative donor (see **Fig. SM2** and the **Supplementary Methods D** for a more detailed discussion). This effectively selects the taxonomically smaller neighbor as the donor. Note that this procedure only approximates the donor group in case of additional evolutionary events or gene tree inference errors influencing the neighboring subtrees' LCAs. In case when both neighbors had different LCAs than their joint LCA, we assumed that the fungal subtrees had an undetermined origin and discarded it from the analysis. Such situations can arise in complex evolutionary scenarios with multiple transfers.

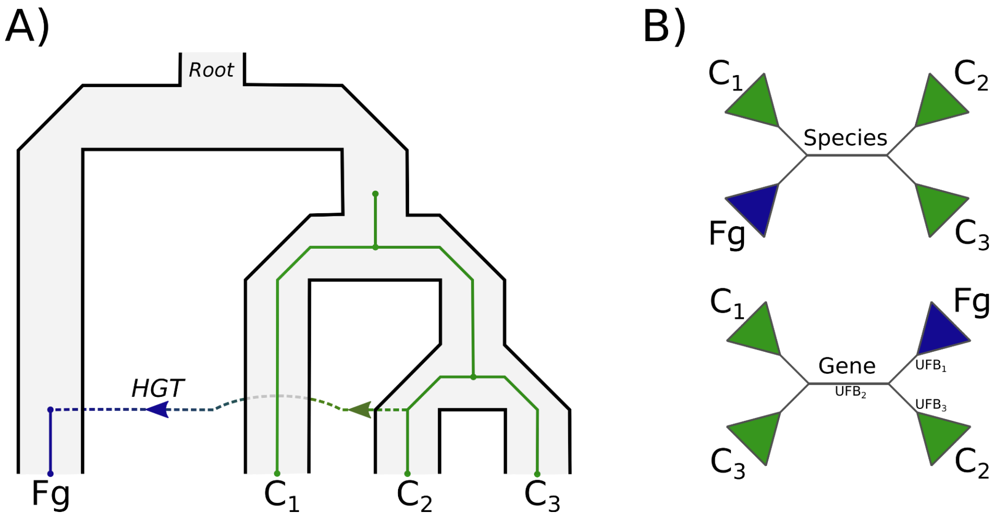

Fig. SM2. An example of a displaced fungal subtree indicating a horizontal gene transfer. A) A species tree with an embedded gene tree, with a single horizontal gene transfer. B) The same species tree (top) and gene tree (bottom) in their unrooted versions. The fungal subtree {Fg} branches with a {C1} and a {C2, C3} subtree in the species tree, and with a {C2} and a {C1, C3} subtree in the gene tree, indicating a different location of the {Fg} subtree in both trees caused by a horizontal gene transfer into the fungal clade. The correct location of the fungal subtree {Fg} in the gene tree is given by the branch adjacent to the {C1} subtree. The subtree {C2} can be inferred as the donor group because the species LCA of {C1, C3} is the same as the species LCA of the whole neighborhood {C1, C2, C3} (see the Supplementary Methods for a detailed discussion). Trees with an average of the three Ultrafast Bootstrap supports UFB_1, UFB_2 and UFB_3 below 90% are discarded in this study.

B) Avoiding false positives in high-throughput HGT studies

There are numerous approaches to HGT detection with different scopes of applications and error rates. Phylogenetic methods, based on comparing the location of the organism of interest in the gene and the species tree of a homologous family, are considered to be the golden standard of HGT inference [1,2]. However, obtaining a gene tree is a complex procedure consisting of several steps which, in a high-throughput setting, are difficult to control and error-prone. An error in any of those steps - such as spurious homology, low-quality alignment, or imperfect tree inference - may lead to differences between the gene and the species tree and result in a false positive HGT event. During the development and testing of our pipeline, we have concluded that multiple factors can inflate the false positive rate, including:

- Relying on sequence similarity instead of phylogenetic trees. Although a useful proxy, the most similar sequence may not be the evolutionarily closest one. This is especially problematic when the molecular clock assumption is violated [3],as well as in the case of multi-domain proteins and the products of gene fusion, which, as shown in this article, is commonly associated with HGT. In this case, the overall sequence similarity is the product of several evolutionary histories. As a consequence, methods based solely on sequence similarity may give improper or inconsistent results (see also [4]), and methods based on phylogenies are preferred.
- Excessively long or short sequences. Both types result in poor-quality alignments and therefore in unreliable gene trees, especially when sequences of vastly different lengths are in the same cluster. Long sequences may additionally result in spurious homology, or contain regions homologous to different sets of proteins.
- Viral and environmental sequences. The placement of such sequences on the species tree is typically unknown, and their location cannot be meaningfully compared unless specific, custom-made species trees are used. Note, however, that databases like NCBI

Taxonomy contain viruses and, when queried for species trees containing them, will return viral taxa placed as an outgroup without warning. As a consequence, if sufficient care is not taken to analyze viral sequences, typical programs will label them as bona fide HGTs.

- The choice of the clustering algorithm. Since xenologs may be distantly related to their donor homolog, the clustering algorithm needs to achieve sufficient sensitivity (in the sense of ability to detect their shared ancestry), but also specificity (in the sense of avoiding clustering unrelated sequences). However, in most types of computational analyses, there is a tradeoff between those two measures, and no algorithm can attain perfect sensitivity and specificity at the same time. Instead, the optimal algorithm for a given task is the one that finds an appropriate balance between specificity and sensitivity. In our case, using the high-specificity, low-sensitivity cd-hit algorithm (-c 0.7 -n 5) in our pipeline resulted in only 129 well-supported HGT events This was caused by the fact that cd-hit was unable to detect homology between more distantly related proteins, and, as a consequence, the clusters were overly fragmented and most of them contained proteins from less than 4 organisms, too few to conduct phylogenetic analysis. On the other hand, using the high-sensitivity, low-specificity mmseqs2 clustering algorithm (easy-linclust) resulted in highly unstable results, as the removal of a small subset of sequences resulted in global changes in the structure of clusters and, in consequence, different sets of HGT events. Furthermore, *mmseqs2 often clustered together unrelated sequences, which we have detected by looking at FASTA files of randomly selected clusters. By analyzing the distribution of the cluster sizes, manually inspecting FASTA files of 20 randomly selected clusters during the development of the pipeline, and analyzing the results of the completed pipeline,* we have concluded that, in the context of this study, the psi-cd-hit and the MCL algorithms offer the best sensitivity-to-specificity ratio.

- Low HSP coverage in the BLAST-based MCL clustering. As mentioned in Supplementary Methods A, during the development of our pipeline, we have inspected two approaches: either just 50% of the query, as in (Keeling et al., 2022), herein referred to as a “query coverage filter”, or 50% of both the query and the subject, referred to as a “two-way coverage filter”. The query coverage filter initially returned more HGT candidates (5393, compared to 3387 for the two-way filter, for the first round of clustering, step (6) of the pipeline). However, those clusters were often large and complex, and, as a consequence, after the filtering of the clusters and the second-round clustering, the query-based filter resulted in fewer candidates (815 compared to 2394 for the two-way filter, step (8) of the pipeline). Most importantly, after a manual inspection of clusters obtained with the query cover filtering, we have found multiple highly incorrect ones. This was caused by the fact that MCL operates on E-values between proteins, and the fact that, due to frequent gene fusions associated with HGT, some proteins had regions homologous to multiple protein families. In MCL-based clustering, such proteins acted as “bridges" that merged unrelated protein families into a single cluster. For those clusters, the multiple sequence alignment consisted of distinct blocks corresponding to those different families, and was unsuitable for phylogenetic tree inference. Furthermore, we have found proteins with mostly fungal homology, but also containing small regions homologous to bacteria, most likely originating from a post-transfer gene fusion. These regions, because of their low E-values, resulted in adding multiple bacterial sequences to the fungal clusters. In our framework, the presence of such regions could wrongly suggest that the whole fungal protein is a xenolog and distort our results. The two-way filter allowed for discarding such proteins, ultimately resulting in the detection of more HGTs and clearer phylogenetic and statistical signals in the data.
- Methods relying on rooted gene trees. Automatic gene tree rooting methods rely on several assumptions that are difficult to meet on the evolutionary distances encountered

in large-scale studies of HGT, such as the molecular clock assumption for the midpoint method or the knowledge of duplication and transfer rates for the DTL method. When those assumptions are violated, automatic rooting methods are inaccurate and inconsistent [6,7].

- Using reconciliation algorithms with improper event weights. Any incongruence can be explained either as a gene transfer or a duplication and multiple losses, depending on the user-specified parameters which reflect the rates of those events. As we have shown in this work, transfer rates differ significantly between fungal lineages, preventing us from using reconciliation in large-scale studies. When we tested the default event weights on midpoint-rooted trees during the initial phase of the development of our pipeline, the PFAM domain composition of the set of putative xenologs closely reflected the proteome background (i.e. the numbers of identified domains of each type had a similar distribution in both groups of proteins; in particular, the regulatory WD40 and Pkinase domains were the most common domains in the putative HGTs), indicating that the gene trees were classified essentially at random. We remark, however, that this test was done before adopting additional filtering criteria, including the two-way HSP coverage filter. As a consequence, the input gene trees were of lower quality than in the final version of the manuscript, which may have contributed to the results of rooting and gene reconciliation methods.
- Complex gene trees with multiple fungal clades. Unless such trees are manually curated, the reconstructed evolutionary scenario is highly unstable, and the evidence for any particular transfer is less supported. In a large-scale study, it is difficult to determine whether multiple clades of fungi arise due to a horizontal transfer or simply an error in tree inference such as the long branch attraction. This is particularly the case for transposons, whose evolutionary histories are particularly complex.

Those considerations have led us to focus on medium-length protein sequences and gene trees with a single fungal clade displaced with respect to its location in the species tree. Despite stringent requirements, our methodology has recovered several previously reported HGTs. Out of numerous xenologs reported in Neocallimastigomycota in (Wang et al. (2019), we have identified 10 as strongly supported. Out of 811 xenologs reported in *Basidiobolus* in (Tabima et al. (2020), we have identified 7 as strongly supported, 23 as weakly supported and 6 in a congruent location in the gene tree.

On the other hand, our goal to limit the number of false positive results necessarily means that we also lose many true HGT events. Several previously reported xenologs failed to pass one or more of our requirements, such as xenologs reported in *R. irregularis* (Li et al. 2018), which we found to be in a congruent location in the gene tree (e.g. EXX72150.1), numerous xenologs reported in *B. dendrobatidis* such as adenylate cyclase (XP_006679093.1) or chitinase (XP_006682460.1) (B. Sun et al. 2016), which we found to be widespread across chytrids, or octin in Phycomyces (XP_018295118.1) (T. A. Nguyen et al. 2018), which we found to be widespread across fungi. The transfer of NRPS to *Mortierella* from *Mycoavidus* (Wurlitzer et al. 2021b) was discarded due to excessive sequence length, although we have identified parallel transfers of other proteins. The transfer of proteins with Gal_Lectin domain to Neocallimastigomycota (Wang et al. 2019) was discarded due to lack of homology to non-fungal species. The transfer of an ATPase to *R. irregularis* (ESA12330.1) (Li et al. 2018) was discarded due to low sequence coverage of the homologous region. Discarding long sequences has also resulted in omitting some known transfers, such as a malpibaldin synthetase from *Mortierella alpina*, a 5541 amino acid-long protein (QOW41314.1). The requirement of a single fungal taxonomic family means that most detected transfers come from distantly related donors. In particular, our pipeline is unable to detect a massive transfer from a fungal parasite *Parasitella parasitica* to its host *Absidia glauca*, since both the donor and recipient are fungal species, and

may have a limited sensitivity towards transfers of those animal genes which already have a fungal homolog. Those kinds of transfer require further studies, presumably with methods designed specifically for each scenario.

As opposed to exploratory studies, focusing on low numbers of uniformly sampled observations is appropriate for comparative statistical analyses. Although the rates and patterns of HGT have been studied in depth in individual lineages of early diverging fungi, such as Neocallimastigomycota (Wang et al. 2019), *Basidiobolus* (Tabima et al. 2020) and *Batrachochytrium* (B. Sun et al. 2016), these studies were exploratory in nature rather than comparative, investigating in depth the potential impact of HGT on a selected lineage. Furthermore, each of those studies employed its own methodology and reported vastly different numbers of xenologs for their respective organisms, making comparative analyses impossible. To our knowledge, the work presented in this manuscript is the first one that applies a unified methodology to multiple fungal lineages in order to provide an extensive comparative study across the tree of life of early diverging fungi. This allowed us to show, for example, that Neocallimastigomycota are more receptive to HGTs than Basidiobolus despite the latter having more HGTs reported in the literature, and more receptive to HGTs than *Linnemania elongata* despite having similar numbers of xenologs in their proteomes. The sample of xenologs obtained with our methodology was sufficient to characterize several patterns with statistical significance.

Our considerations regarding the design of a high-throughput horizontal gene transfer detection pipeline draw a parallel to the *no free lunch* theorems from mathematical optimization, which state that no single algorithm is suitable for all optimization problems [9]. In particular, studies of inter- and intra-kingdom HGT seem to have different requirements for their algorithms, just as studies of horizontal transfer of transposon or viral sequences, which have particularly complex gene trees. A single pipeline is therefore mostly suitable for a single type of HGT events, and may give spurious results when applied elsewhere.

C) Controlling long branches in gene trees

Evolutionarily distant sequences can make the topology of a phylogenetic tree unreliable despite high support values (Rodríguez-Ezpeleta et al. 2007). These include distant groups of sequences, visible in the tree as long internal branches. To avoid topological errors caused by distantly related sequences, we have adopted a strategy of iteratively splitting trees into two by cutting long branches until all remaining branches are no longer than a given threshold.

Consider an unrooted binary tree as $T = (V, E)$, i.e. an undirected acyclic graph for which each node has a degree equal either 1 or 3. With each branch e we have an associated branch length parameter denoted as e.length. Starting from a forest with a single unrooted tree $F_0 = \{T\}$, we want to find a sequence of edge cuts that results in a forest $F = \{T_1, T_2, \ldots, T_k\}$ such that each edge of each T_i is shorter than a given threshold. Formally, we define cutting an edge $e = (v_1, v_2)$ of a tree T in forest F as removing the edge e and contracting any resulting degree-2 nodes (including summing the lengths of their adjacent branches).

Obtaining the desired forest can be accomplished by iteratively cutting a single branch with length over the threshold and updating branch lengths. Algorithm 1 shows the details of our approach. It is not difficult to show that the graph inferred by Algorithm 1 is a forest of unrooted trees. In addition, the forest does not depend on the order of long edges removals as long as α is fixed. From the computational complexity point of view, the algorithm can be implemented to run in $O(|T|)$ time, where $|T|$ is the size of the input tree.

Algorithm 1.

Input: An unrooted tree $T = (V, E)$; A branch length threshold α.

Output: A forest obtained from cutting T's edges, with all edges no longer than α.

While there exists an edge $e \in E$ with *e*.length >= α:

Let $\{v, w\} = e$

Remove *e* from *E*

For *u* in $\{v, w\}$:

If *u* is internal:

Identify nodes u_1, u_2 adjacent to *u*

Create edge $e_u = \{u_1, u_2\}$

Set e_u.length = $\{u_1, u\}$.length + $\{u, u_2\}$.length

Add e_u to *E*

Remove *u* from *V*

Return (V,E)

D) Detecting horizontal gene transfer in unrooted gene trees

Motivation. The most reliable evidence for a horizontal gene transfer is based on the comparison of the topologies of two phylogenetic trees: a *gene tree*, representing the evolutionary relationship of a homologous family, and a *species tree*, representing the evolutionary relationships of species. When genetic material is exchanged between species horizontally, the resulting homologs–termed *xenologs*–start to evolve independently. This occurs after the speciation of their host species, and, in consequence, the evolutionary difference is larger for the species than for their xenologs. This leads to a characteristic *incongruence*, i.e. a difference in topology, of the gene and the species trees: the xenologs branch together in their tree, but their host species do not. An example of such incongruence is shown in **Fig. SM3**. The analysis of tree incongruences is one of the basic tools in studying genomic events such as horizontal gene transfers and gene duplications, as well as populational effects, such as deep coalescence (an ancient separation of alleles in closely related organisms).

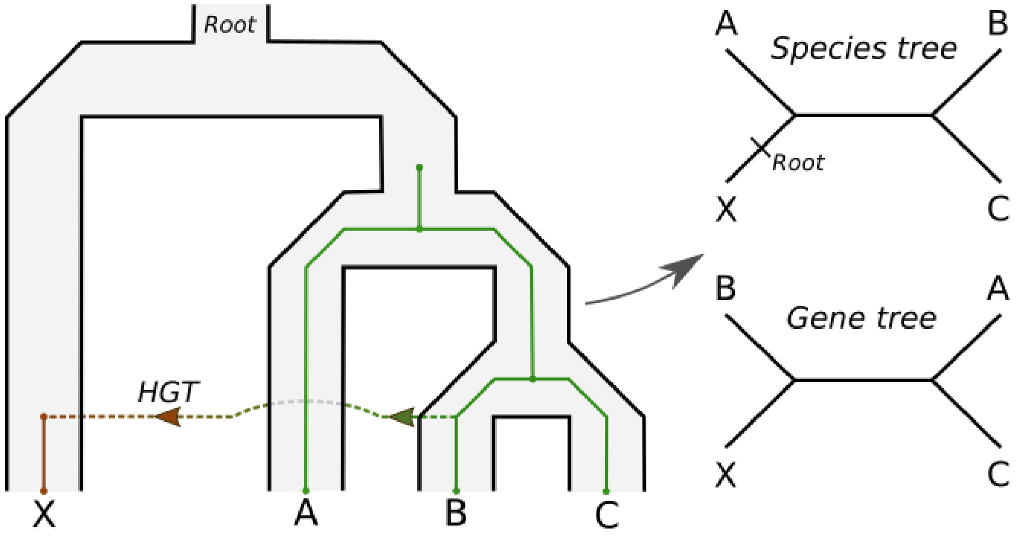

Fig. SM3: The incongruence between trees caused by a horizontal gene transfer. The recipient clade X branches with clades {A} and {B, C} in the unrooted version of the species tree, and with clades {A, C}, {B} in the gene tree.

Arguably, the most developed methodology for studying genomic effects based on tree incongruence is the *tree reconciliation*, in which the gene tree is embedded into the species tree (with possible horizontal branches of the gene tree), in a way that minimizes a weighted sum of the numbers of different evolutionary events. With fine-tuned parameters and accurate input data, reconciliation allows to reconstruct the whole evolutionary history of a homologous family, and is an indispensable tool in detailed phylogenetic studies. On the other hand, its requirements, necessary for a detailed result, limit its applicability in a high-throughput setting. A common version of the method, the DTL model, requires the user to specify a set of weights that tell the method how likely a horizontal gene transfer is to occur in comparison to a gene duplication. However, any incongruence can be explained using only duplications or only transfers. Reliable methods of weight estimation are lacking, and it is unlikely that a single set of weights applies for all species, as the rates of transfers differ highly between them.

Another limiting requirement of tree reconciliation in the DTL model is that the most developed methods rely on the knowledge of the direction of evolution, expressed as the location of the root of the gene tree. However, the typical sequence substitution models used in maximum likelihood method of gene tree inference assume that evolution is time-reversible and result in unrooted trees. Automatic rooting methods are often inaccurate, resulting in improperly placed roots which lead to improperly reconstructed evolutionary scenarios. This adds on top of the unavoidable inaccuracies of tree inference itself. It is therefore difficult or even impossible to obtain sufficiently high-quality input data in a high-throughput setting to get a reliable sample of xenologs using tree reconciliation.

These considerations have led us to develop an alternative approach suited for detecting xenologs in a given set of genomes. In this task, we do not need to reconstruct whole scenarios, as we are only interested in testing whether a particular branch – the one adjacent to the fungal clade – represents a vertical or horizontal inheritance. We have noticed that a particular class of such transfer edges can be reliably detected in unrooted gene trees. Namely, a transfer event results in a shift of the fungal clade in the gene tree with respect to its location in the species tree. An example of such a shift is shown in **Fig. S9**. Therefore, the desired test can be based on a comparison of the locations of the fungal clades in the two trees.

The main difficulty in using this phenomenon to test whether a branch corresponds to a transfer is to rigorously define the notion of a location in a tree, taking into consideration natural differences between the gene and species trees. The latter often contain polytomous nodes, while the former are binary, but usually differ from the corresponding species tree in their overall topology due to other evolutionary events and tree reconstruction errors. Therefore, relying on whole topologies of the two trees to measure the location shift is not desirable.

To circumvent the topological problems associated with the different natures of both trees, we define the location shift using the label composition of the clades neighboring with the fungal clades in both trees. The general idea, formalized in this section, is that the location of a fungal clade is identical in both trees if its neighboring clades can be matched in a way that the sets of leaves of matched clades are identical, i.e. correspond to the same sets of species.

Detecting fungal clades in gene trees. In order to detect displaced (incongruent) fungal clades, we first need to be able to identify the clades themselves. We define an unrooted tree as a pair (V, E) of nodes and edges, where E is a set of two-element sets of nodes. Let G be an unrooted gene tree and S be its corresponding species tree (treated as unrooted in this paragraph), so that the set of species in S is the same as the set of host species of

genes/proteins in *G*. We define a clade as a subtree of *G* or *S* that can be obtained by cutting a single edge and selecting one of the resulting subtrees. The cut edge is referred to as the clade's *attaching edge*, and the attaching edge's node belonging to the clade is referred to as the clade's *root node*.

Let *L* be the set of all fungal leaves (in either *G* or *S*). We say that a clade is induced by *L* if it contains only leaves from *L* and is maximal with respect to containment, i.e. any clade which contains it also contains leaves outside of *L*. In the gene tree, the set *L* may induce more than one clade. In order to analyze the displacement of fungal clades in *G*, we first look for all clades induced by the set of fungal leaves *L* in *G*.

Algorithm 2 shows a procedure to identify all clades induced by a given set of leaves in a given tree. It employs a boolean data structure all_in_L[*w*, *v*] which, for a directed edge (*w, v*), stores a value True if all leaves reachable from *w* through *v* are in the set of leaves *L*, and a value False if any leaf reachable from *w* through *v* is not in *L*. Algorithm 2 first identifies all directed edges whose end is a root node of some (possibly non-maximal) fungal clade. Next, it identifies *splitting edges*, i.e. edges such that there are only fungal species on one of their sides. Finally, the algorithm identifies clade attaching edges as splitting edges adjacent to some non-splitting edges. Note that this procedure works as well for non-binary trees. However, it requires each clade to have its own attaching edge. As a consequence, for non-binary species trees it may return more clades than expected if the species in *L* form a single clade in some, but not all, binarizations of the tree.

```
Algorithm 2.
Input: Unrooted tree G = (V, E); Subset of leaves L.
Output: Directed attaching edges of clades induced by L.
all_in_L = an empty map from pairs of nodes (n1, n2) to bool
clade_edges = an empty list
Function traverse_tree(w, v){
        If v is a leaf:
                If v ∈ L:
                        all_in_L[w, v] = True
                Else:
                        all_in_L[w, v] = False
        Else:
                Get N = a list of v's neighbors other than w
                For each node n in N:
                        If all_in_L[v, n] is undefined:
                                traverse_tree(v, n)
                If all_in_L[v, n] == True for all n ∈ N:
                        all_in_L[w, v] = True
                Else:
                        all_in_L[w, v] = False
}
For each leaf l:
        traverse_tree(l, l.parent)
For each node n:
        Get a list of n's neighbors v_1, v_2, …, v_k
        Get a boolean list b_i = all_in_L[n, v_i] for each i in 1, 2, …, k
        If there exists j such that b_j == False:
                For all i such that b_i == True:
                        Append (n, v_i) to clade_edges
Return clade_edges
```

Detecting clade location shift. Let G be an unrooted gene tree and S be its corresponding species tree, so that the set of species in S is the same as the set of host species of genes/proteins in G. For now, we will consider a case when G has a single fungal clade. Let G_f denote the fungal clade in G and let S_f denote the fungal clade in S. For a given clade C, denote $N(C)$ the set of neighboring clades. Note that since G is binary, we have $|N(G_i)| = 2$ for each i, but we may have $|N(S_f)| >= 2$ if (and only if) S is non-binary. Let $L(T)$ be the set of species corresponding to leaves of the tree T.

Consider a simple case where S is binary. Then, we will say that the fungal clades G_f and S_f have an identical location in both trees if the clades from $N(G_f)$ and $N(S_f)$ can be matched one-to-one with respect to their species composition. That is, if $N(G_f) = \{A, B\}$ and $N(S_f) = \{C, D\}$, we require that either $L(A) = L(C)$ and $L(B) = L(D)$ or $L(A) = L(D)$ and $L(B) = L(C)$. This indicates that the fungal clade has an identical branching pattern in both trees, in the sense that it branches with the same taxonomic clades regardless of their internal topology. However, S may contain multiple neighboring clades, especially when highly polytomous species trees from public databases are used. In this case, we assume that a polytomy represents an unknown order of speciations, and the tree can be binarized in a way that represents the true sequence of speciation events. When comparing sets of leaf labels, a binarization of a polytomous node corresponds to merging clades from $N(S_f)$ into two sets of species. If those sets can be matched with clades from $N(G_f)$, we say that the fungal clade has an identical location in both trees.

These considerations allow us to give a simple test whether the fungal clade is displaced in G with respect to its location in S. Let $N(G_f) = \{N^G{}_1, N^G{}_2\}$, and let $N(S_f) = \{N^S{}_1, \ldots, N^S{}_k\}$. We say that the fungal clade has the same location in G and S if and only if each $L(N^S{}_1)$ is a subset of either $L(N^G{}_1)$ or $L(N^G{}_2)$ for $i = 1, 2, \ldots, k$. This is equivalent to merging clades from $N(S_f)$ into a set corresponding to $N^G{}_1$ and a set corresponding to $N^G{}_2$. It follows that the fungal clade can be considered to be displaced if any $N^S{}_i$ has leaf labels present in both $N^G{}_i$ clades.

However, this kind of displacement can occur not only because of HGT into fungi, but also because of a HGT between species from $N(G_f)$ or a gene tree inference error. In order to differentiate between those cases, we need an additional constraint. Namely, we need to ensure that the gene tree has a branch such that, if G_f was to be repositioned to that branch, it would have an identical location as S_f in S. This means that G contains a location for the fungal clade that would correspond to vertical evolution. Otherwise, G_f would be considered as displaced regardless of its position in G. Checking for this location can be accomplished simply by ignoring fungal leaves in G, iterating over all edges in G, taking splits induced by those edges and checking if they can be matched with $N(S_f)$. Note that the lack of the location of vertical inheritance does not preclude the possibility of HGT, but our method is not suitable to detect such cases.

Consider now the case when fungi induce multiple clades in G, denoted $G_1, \ldots, G_m$. Then, we process each clade independently of the other, ignoring other fungal species. Namely, for each i we take the neighboring clades $N(G_i)$, remove any fungal species from those clades, and compare them with $N(S_f)$ (which, by definition, don't contain any fungal species). Note that checking for the location of vertical inheritance in G does not depend on the number of fungal clades in this tree, and can be done just once.

Identifying the donor group. A fungal clade with an unexpected branching pattern is indicative of a HGT into the fungi from the last common ancestor of one of the neighboring clades. In order to determine which one of the two is the donor one, we analyze their last common ancestors and the last common ancestor of the two clades combined. In the transfer scenarios analyzed in this work, the donor group branches within a larger clade of species (note that this may not be the case for ancient horizontal transfers between the ancestors of two monophyletic clades). A consequence is that one of the clades neighboring with the fungal one in the gene tree, containing species from the aforementioned larger clade, will have the same last common

ancestor (in the rooted version of the species tree) as the two neighboring clades combined. For example, in **Fig. S9**, the last common ancestor of the {A, C} clade is identical to the last common ancestor of the two neighboring clades combined into {A, B, C}, and the last common ancestor of the {B} clade is below it. In this work, we identify the donor clade as the one for which the common ancestor is below the common ancestor of the combined clades. If both neighboring clades in the gene tree have an identical last common ancestor, we also treat it as the donor. If neither case is met, we discard the tree as either likely containing inference errors or with an evolutionary scenario too complex for an automated analysis.

References

1. Richards TA, Leonard G, Soanes DM, Talbot NJ. Gene transfer into the fungi. Fungal Biology Reviews. 2011. pp. 98–110. doi:10.1016/j.fbr.2011.04.003

2. Ravenhall M, Škunca N, Lassalle F, Dessimoz C. Inferring horizontal gene transfer. PLoS Comput Biol. 2015;11: e1004095.

3. Stadler PF, Geiß M, Schaller D, López Sánchez A, González Laffitte M, Valdivia DI, et al. From pairs of most similar sequences to phylogenetic best matches. Algorithms Mol Biol. 2020;15: 5.

4. Richards TA, Leonard G, Soanes DM, Talbot NJ. Gene transfer into the fungi. Fungal Biology Reviews. 2011. pp. 98–110. doi:10.1016/j.fbr.2011.04.003

5. Capella-Gutierrez S, Silla-Martinez JM, Gabaldon T. trimAl: a tool for automated alignment trimming in large-scale phylogenetic analyses. Bioinformatics. 2009. pp. 1972–1973. doi:10.1093/bioinformatics/btp348

6. Wade T, Rangel LT, Kundu S, Fournier GP, Bansal MS. Assessing the accuracy of

phylogenetic rooting methods on prokaryotic gene families. PLoS One. 2020;15: e0232950.

7. Lamarca AP, Schrago CG. Fast speciations and slow genes: uncovering the root of living canids. Biological Journal of the Linnean Society. 2020. pp. 492–504. doi:10.1093/biolinnean/blz181

8. Alexander WG, Wisecaver JH, Rokas A, Hittinger CT. Horizontally acquired genes in early-diverging pathogenic fungi enable the use of host nucleosides and nucleotides. Proc Natl Acad Sci U S A. 2016;113: 4116–4121.

9. David H. Wolpert and William G. Macready, “No Free Lunch Theorems for Optimization” IEEE Transactions on Evolutionary Computation Vol.1 No.1 pp.67-83,1997. Journal of Japan Society for Fuzzy Theory and Systems. 1997. pp. 696–697. doi:10.3156/jfuzzy.9.5_696
